# Biodegradable Gelatin Microcarriers Facilitate Re-Epithelialization of Human Cutaneous Wounds - An *In Vitro* Study in Human Skin

**DOI:** 10.1371/journal.pone.0128093

**Published:** 2015-06-10

**Authors:** Susanna Lönnqvist, Jonathan Rakar, Kristina Briheim, Gunnar Kratz

**Affiliations:** 1 Division of Experimental Plastic Surgery, Department of Clinical and Experimental Medicine, Faculty of Health Sciences, Linköping University, Linköping, Sweden; 2 Center for Integrative Regenerative Medicine (IGEN), Department of Clinical and Experimental Medicine, Linköping University, Linköping, Sweden; 3 Department of Hand and Plastic Surgery, Region Östergötland, Linköping, Sweden; Institute for Frontier Medical Sciences, Kyoto University, JAPAN

## Abstract

The possibility to use a suspended tridimensional matrix as scaffolding for re-epithelialization of *in vitro* cutaneous wounds was investigated with the aid of a human *in vitro* wound healing model based on viable full thickness skin. Macroporous gelatin microcarriers, CultiSpher-S, were applied to *in vitro* wounds and cultured for 21 days. Tissue sections showed incorporation of wound edge keratinocytes into the microcarriers and thicker neoepidermis in wounds treated with microcarriers. Thickness of the neoepidermis was measured digitally, using immunohistochemical staining of keratins as epithelial demarcation. Air-lifting of wounds enhanced stratification in control wounds as well as wounds with CultiSpher-S. Immunohistochemical staining revealed expression of keratin 5, keratin 10, and laminin 5 in the neoepidermal component. We conclude that the CultiSpher-S microcarriers can function as tissue guiding scaffold for re-epithelialization of cutaneous wounds.

## Introduction

The use of scaffolds for dermal regeneration in combination with cultivated keratinocytes is well accepted [1,2]. Several kinds of medical device products based on allogeneic and xenogeneic ECM components aimed to promote wound healing have been introduced [3]. The desired qualities for a provisional wound healing matrix include biocompatibility, durability, a biomimetic surface for resident cells and support for tissue regeneration [4]. However, most of these products are of a static nature meaning they must be produced in exactly the shape of the defect which is often clinically inefficient. This motivates the introduction of a malleable scaffold that is optimized for culture of human cells, biodegradable without toxic residues and that stabilizes when applied to a defect. These requirements can be met with the application of macroporous spherical microcarriers [2]. Culturing of cells on microcarriers has primarily been a method for high-density cell expansion [5] and as a delivery vehicle for transplantation of attached cells [6]. We have previously investigated the use of microcarriers as cell delivery tool and as injectable scaffolding for dermal regeneration. Seland et al. showed that when rat skin wounds were grafted with keratinocyte-seeded microcarriers the healing resulted in a significantly thicker neoepidermis after 16–21 days [7]. Work by Huss et al. showed the microcarriers to function as scaffolding for ingrowth of dermal fibroblasts when injected into the dermis, resulting in a vascularized neodermis after carrier absorption [8].

Gelatin is a well-tolerated substance with low antigenicity [9] and has been recognized as a useful material for skin wound therapies [10]. Derived from collagen, gelatin shares many of its biological properties with added benefit of increased accessibility and ease of molecular modifications. The clinically approved CultiSpher-S microcarrier (Percell Biolytica AB, Åstorp, Sweden) consists of highly cross-linked porcine derived type A gelatin. The diameter of the spherical carrier ranges between 70 and 170 μm with internal pore size between 10 and 20 μm. There is a large body of research (reviewed in [2]) behind this particular microcarrier type and a need to further investigate its use in wound healing applications. Agglutination of gelatin-based microcarriers occurs when they are transferred to a wound environment, rapidly forming a stable compliant porous matrix. Porosity is an important factor for the survival and infiltration of cells and therefore crucial for the utility of a cell-carrier [11]. Reciprocity of modulation between cells in the wound environment and the ECM is important for normal wound healing [12] and the porosity and structure of the microcarriers creates a supportive biomimetic scaffold.

Several *in vitro* and *in vivo* studies have revealed that a variety of cell lines exhibit survival and proliferation when cultured on CultiSpher-S microcarriers [5]. In the study by Huss et al. partial absorption of the CultiSpher-S was evident after eight weeks when injected into the human dermis and complete absorption occurred after 26 weeks. The rate of degradation is dependent on where the microcarriers are implanted and the tissue activity. The injection of CultiSpher-S into the human dermis evoked no inflammatory reaction or capsulation [8].

Based on the previous reports supporting the use of CultiSpher-S in wound healing and work performed by the group on injectable microcarriers as dermal scaffolding we have investigated the use of these microcarriers on re-epithelialization without additional cell transplantation. There is a need to evaluate the efficacy of these microcarriers for use in guided tissue regeneration without cellular pre-seeding [8]. The present study was performed in a human in *vitro model* for wound healing. Tissue culture wound models are of value for investigating the wound healing process in human skin and a large number of identical wounds *in vitro* enables a more rapid discovery process and supports planning of subsequent *in vivo* studies [13]. Microcarriers were applied to *in vitro* wounds inflicted in excised human skin and the wounds were cultured for 21 days. The effect of the CultiSpher-S microcarriers was evaluated by studying the ingrowth of wound edge keratinocytes into the microcarriers and the formation of a neoepidermis.

## Material and Methods

### Tissue culture and sectioning

Human skin samples were rescued from tissue discarded during routine abdomenoplasty and de-identified to comply with Swedish ethical regulations governing the use of human materials. Skin was processed within 12 hours. Wounds extending over the dermo-epidermal junction were prepared as previously described [13]. In brief, skin was washed in PBS and adipose tissue was removed. Wounds were inflicted in 6 mm biopsies with a 3 mm biopsy punch (Integra Milltex, York, PA). Microcarriers were hydrated, sterilized by autoclaving and washed according to instructions from manufacturer (PerCell Biolytica, Åstorp, Sweden). Addition of microcarriers to wounds was carried out by depositing 10 μl of 20 mg/ml microcarrier solution, with a cut off pipet tip, directly to the wound bed. Experimental wounds received microcarrier suspension and were cultured in maintenance medium consisting of DMEM supplemented with 10% fetal calf serum (FCS), penicillin 50 U/ml, and streptomycin 50 μg/ml (Gibco, Life technologies, Paisley, UK). Control wounds did not receive microcarriers and were cultured in maintenance medium. Wounds were cultured in individual wells in 24 well plates (BD Bioscience, Franklin Lakes, NJ, US). Six experimental wounds and six control wounds were prepared and the experiment was performed twice. All wounds were cultured with the wound area free from maintenance medium for the first 48 hours to allow microcarriers to sediment in the wound bed, after which the wounds were submerged.

To investigate the effect of gelatin in solution in the wounds, six wounds were cultured with addition of 2% gelatin (Sigma-Aldrich, St. Louis, MO). Gelatin solution was deposited directly in the wound area and wounds were left free of maintenance medium for 48 hours as described above, and then cultured submerged in medium for the remaining culture time.

Additionally, experimental and control wounds were treated as mentioned above but cultured submerged in maintenance medium for the first 14 days of culture after which the wounds were air-lifted using cell culture inserts (BD Bioscience, Franklin Lakes, NJ, US) for seven days to observe stratification of the neoepidermis.

All wounds were collected after 21 days of culture and snap-frozen in liquid nitrogen. Specimens were mounted to sample holder with OCT (optimal cutting temperature compound, HistoLab, Gothenburg, Sweden) and sectioned in 10 μm and 50 μm sections with a Leica 3050CM cryostat (Leica Microsystems, Wetzlar, Germany).

### Hematoxylin-eosin staining and immunohistochemistry

Sections were air dried for 30 minutes before washing in PBS and subsequently stained with routine Mayer’s hematoxylin and eosin (HistoLab). Sections were mounted for microscopy with Mountex mounting medium (HistoLab).

For immunohistological staining procedures sections were air dried for 60 minutes and fixated with 4% paraformaldehyde or acetone. Sections were washed in PBS and blocked with bovine serum albumin (BSA) at 2.5% for 30 minutes. For staining of 10 μm sections incubation with primary antibody took place in a humidified chamber for 60 minutes, for 50 μm sections at 4° C overnight. Primary antibodies were used at the following dilutions; pancytokeratin AE1/AE3 1:200 (Ms Mab MAB3412, Chemicon, Millipore, Billerica, MA), keratin 5 1:200 (Rb Mab 04–587, Millipore), keratin 10 1:400 (Ms Mab MAB3230, Millipore), laminin 5 1:100 (Ms Mab MAB1947, Millipore) and Ki-67 1:200 (Ms Mab MAB4190, Millipore). Secondary anti-mouse or anti-rabbit antibodies conjugated with Alexa Fluor 488 or Alexa Fluor 546 (Invitrogen, Life technologies, Paisley, UK) were applied for 60 minutes in a humidified chamber. After washing and draining, sections were mounted with ProLong Gold mounting medium (Molecular Probes, Life technologies, Paisley, UK) containing 4, 6-diamidino-2-phenylindole (DAPI).

### Image analysis and statistical analysis

Stained sections were visualized with an Olympus BX41 light/fluorescence microscope and images captured using an Olympus DP70 CCD camera (Olympus, Solna, Sweden). Thickness of the neoepidermis in experimental and control wounds was measured digitally. The wound bed area of interest was delimited by tracing the base of the wound. Monochrome thresholding was applied to pancytokeratin staining (Alexa Fluor 546 red) and an average height of neoepidermis across the wound base was calculated. Three tissue sections per wound were included in the measurements. Measurements were performed in Fiji [14] and results are reported as average thickness (μm ± SEM) of re-epithelialization. Statistical comparisons were made using t-test in GraphPad Prism 5 (GraphPad Software, La Jolla, CA) with p < 0.05 considered significant. Confocal images were captured with Zeiss LSM 700 microscope (Carl Zeiss AG, Oberkochen, Germany) using Zeiss ZEN software.

## Results

To investigate the effects of CultiSpher-S as a gelatin scaffold on the re-epithelialization of cutaneous wounds the microcarriers were applied to *in vitro* wounds. Wounds were cultured in DMEM supplemented with 10% FCS for 21 days. Five experimental wounds out of twelve showed complete re-epithelialization and incorporation of microcarriers, and where microcarriers were not completely retained almost complete or partial ingrowth into the remaining carriers could be observed. The partially re-epithelialized experimental wounds were included in the thickness measurements.

**Fig 1:**
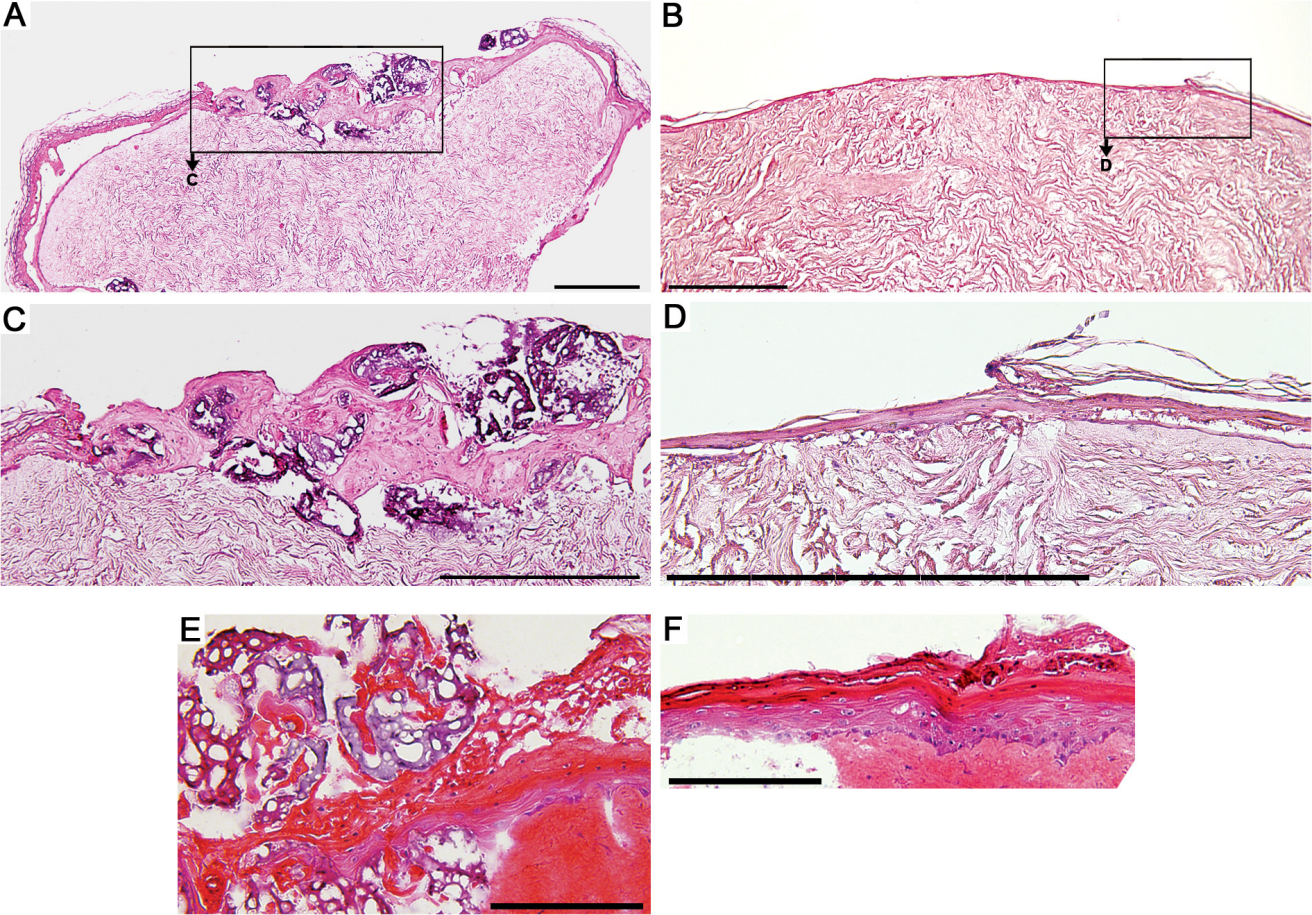
Representative images of hematoxylin-eosin staining of *in vitro* wounds. (A) Section of re-epithelialized wound bed with incorporated microcarriers; (B) section of re-epithelialization of control wound. (C and D) Magnifications of wound edge of (A) and (B). Scale bar A-D = 500 μm. (E) Section of wound cultured submerged in culture medium for 14 days and air-lifted for seven days with microcarriers; (F) Wound edge of air-lifted control wound. Scale bar E-F = 200 μm.

Hematoxylin-eosin staining of cryosectioned tissue showed microcarriers incorporated in the re-epithelialized wound bed (Fig 1A and 1C). Control wounds re-epithelialized fully as expected and showed complete neoepidermal coverage (Fig 1B and 1D). Air-lifting of wounds was carried out for the last seven days of a 21-day culture in DMEM supplemented with 10% FCS. Air-lifting of wounds resulted in stratification of the neoepidermis enclosed in and around the microcarriers. Control wounds showed an expected degree of stratification (Fig 1 E and 1 F). The microcarriers adhered to the wound bed without gap formation (S1 Fig).

**Fig 2:**
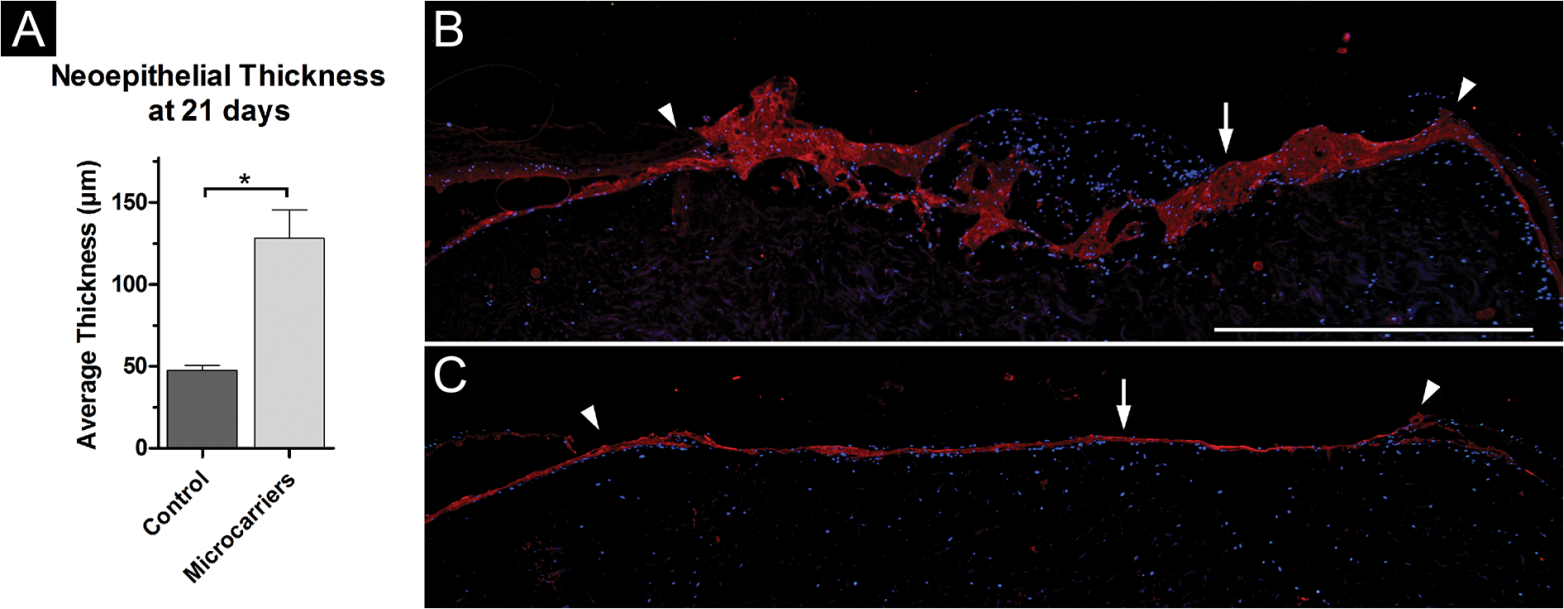
Measurement of neoepithelial thickness. (A) Average thickness of neoepidermis in wounds cultured with CultiSpher-S microcarriers (n = 15) compared to control wounds (n = 11; asterisk indicates significance, p < 0.05). (B) Immunohistological staining with antibodies against cytokeratin (red) of wound cultured with microcarriers; arrow indicates neoepidermis, arrowheads indicate wound edges. (C) Immunohistological staining with antibodies against pancytokeratin of control wound; arrow indicates neoepidermis, arrowheads indicate wound edges. Scale bar = 1000 μm for (B) and (C).

Wounds treated with microcarriers showed a significantly thicker neoepidermis (128.0 μm±17.4) on average compared to control wounds (47.6 μm±3.09) (Fig 2A, S1 Datatable and S1 Imageset). Sections were stained with pancytokeratin antibodies to determine the epithelial origin of the ingrown cells. The cytokeratin staining in red in Fig 2B and 2C extends the complete wound bed in both the experimental wound (B) and the control wound (C) exhibiting a thin neoepidermis. Nuclear staining with DAPI reveals cell nuclei present in the middle of the experimental wounds in and on the CultiSpher-S microcarriers.

Wounds cultured with 2% gelatin solution did not re-epithelialize fully over the culture time of 21 days but the neoepithelial tongue closest to the wound edge was observed to be thicker than in normal controls. Additionally, single cells and clusters of cells could be observed dispersed over the length of the wound bed (S2 Fig).

**Fig 3:**
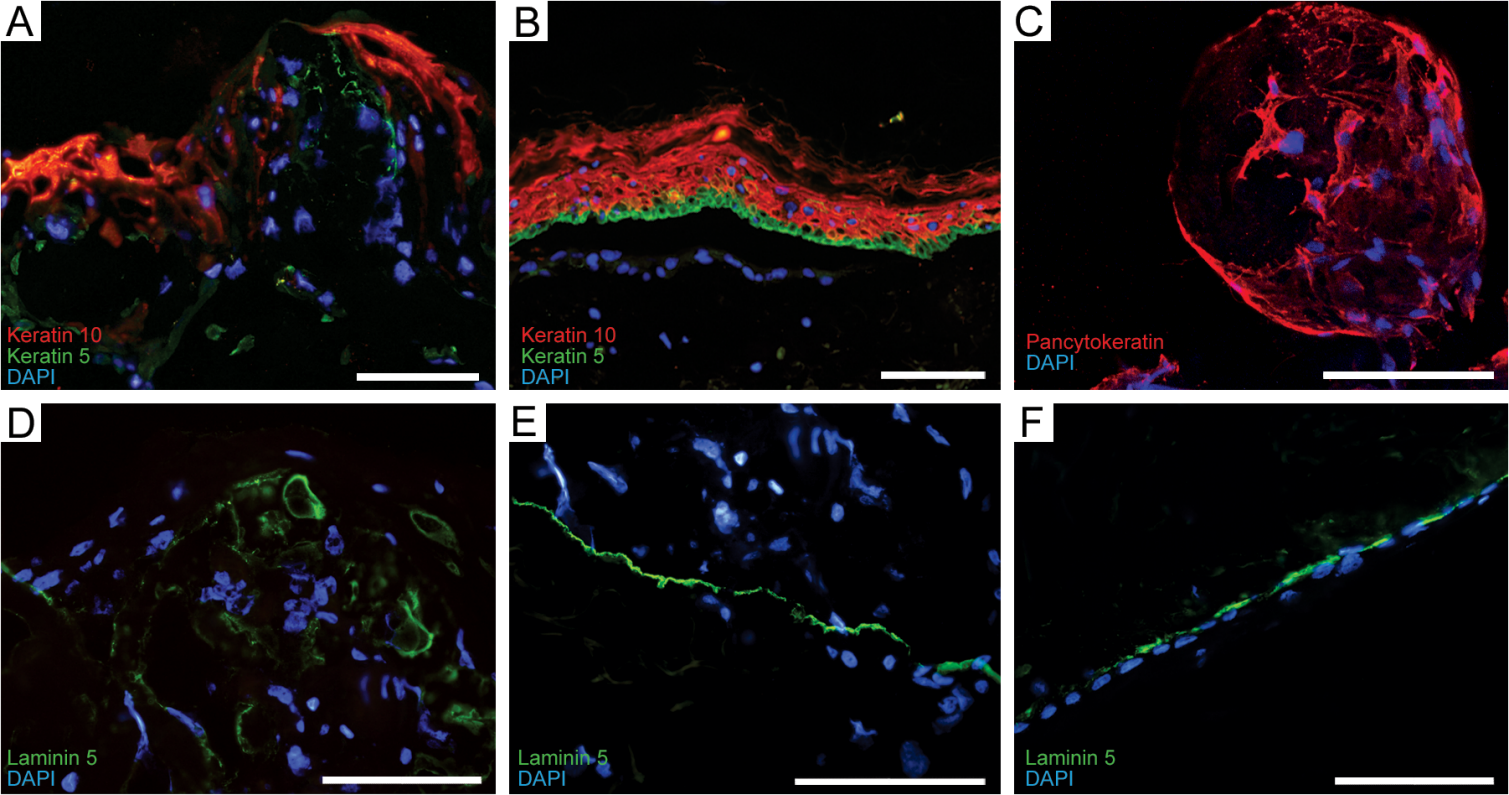
Immunohistochemical staining. (A) Immunohistological staining against keratin 10 (red) and keratin 5 (green) in neoepidermis with incorporated microcarriers and compared to (B) normal control skin. (C) Immunohistological staining against pancytokeratin (red) showing microcarrier populated by keratinocytes. (D) Immunohistological staining against laminin 5 (green) in neoepidermis with microcarriers, (E) unwounded skin, and (F) neoepidermis of control wound. Nuclear staining with 4, 6-diamidino-2-phenylindole (DAPI). All scale bars = 100 μm.

Immunohistological staining revealed expression of keratin 5 and keratin 10 in the neoepidermis of wounds with microcarriers (Fig 3A) in compartments corresponding to mature epidermis of normal skin (Fig 3B). Local expression of laminin 5 in keratinocytes on the surface of the microcarriers (Fig 3D) was detected, compared to the uniform expression in unwounded skin (Fig 3E) and neoepidermis of control wound (Fig 3F). No increase in Ki-67 expression at wound edges could be detected (not shown). Confocal imaging of pancytokeratin staining of wounds with microcarriers showed the CultiSpher-S completely populated by keratinocytes (Fig 3C).

## Discussion

We investigated the effects of providing resident cells in cutaneous *in vitro* wounds a porous scaffold in the form of CultiSpher-S gelatin microcarriers. We utilized an *in vitro* wound healing model based on viable human skin in culture. The tissue culture model provides a tool for investigating healing of standardized wounds over a time span of several weeks with cells present in the biopsy in their native environment within the tissue biopsy. Avoiding animal studies is always desirable and using a model based on the target tissue enables investigations of the complex process of wound healing in a more contextual manner than by utilizing cell assays. Previous work by Seland et al. [7] reporting thicker neoepidermis in wounds grafted with keratinocyte-seeded microcarriers was performed in athymic rats. The aim of the present study was to investigate the effects of adding gelatin microcarriers only to deep dermal wounds. A limitation of the *in vitro* wound healing model is the lack of a systemic response of the vascular and immune systems. However, the process of foremost interest for the study was re-epithelialization. This implies activation, proliferation and migration of wound edge keratinocytes and these responses to wounding are present in the model, visualized by re-epithelialization of standardized wounds within a delimited time. We measured re-epithelialization and observed epidermal regeneration. In our experimental setup the wounds are cultured for three weeks to allow re-epithelialization under normal conditions. Wounds with microcarriers presented re-epithelialization with visible microcarriers present in the wound bed. This indicates that the microcarriers are stable enough to provide scaffolding for the keratinocytes over a time-span required for successful wound healing.

The porous structure of the microcarriers enables cell migration into the carriers, creating an open scaffold for wound edge keratinocytes to cover during the re-epithelialization process. Wound edge keratinocytes populated the microcarriers and re-epithelialized the wound with a thicker epidermal component compared to control wounds. When staining with antibody against Ki-67, a proliferation marker, no increase in cells expressing the antigen could be found at the wound edges indicating that the main event leading to microcarrier incorporation is migration rather than proliferation. The epithelial origin of the populating cells was confirmed by immunohistochemical staining using a mixture of antibodies that detect both acidic and basic keratins (pancytokeratin) [15]. Confocal imaging revealed that keratinocytes populated not only the surface of the microcarriers but also the centers of the spheres and this was evident in all cross-sections of microcarrier-seeded wounds.

The incorporation of microcarriers resulted in a disorganized appearance of the neoepidermis. To establish the organization we investigated the hallmarks of a regenerated epidermis, which are the terminal differentiation of keratinocytes [16] and the presence of a mature dermo-epidermal junction [17]. Immunohistochemical staining of sections of the *in vitro* wounds with antibodies against keratin 5 and 10 was performed. Keratin expression by keratinocytes is compartmentalized and dependent on state of differentiation. Basal keratinocytes express keratins 5 and 14 [18,19]. Upon terminal differentiation and extrusion from the basal niche, suprabasal keratinocytes express mainly keratins 1 and 10 [19]. Immunohistochemical staining revealed expression of both keratin 5 and 10 in the wound bed of wounds with neoepidermis incorporated in microcarriers. The localization of the expression of the two keratins mimics normal mature epidermis with expression of keratin 10 in the upper layers of the neoepidermis and positive staining of keratin 5 in lower layers. This indicates an organization similar to normal epidermis despite the presence of microcarriers.

The wound bed with incorporated CultiSpher-S microcarriers showed positive staining for the major basal membrane component laminin 5. The dermo-epidermal junction is a complex network of proteins, and laminins function as key components in the anchoring complexes that ensure communication between the two separate tissue compartments [20]. A stable basal membrane is vital to skin barrier stability [21]. The discontinuous expression of laminin 5 observed in the epidermis of wounds with microcarriers appears to be on the surface of the carriers. This is histologically different from the normal organization of the basal membrane but likely explained by the topology of the carrier scaffold. The microcarriers were added to the experimental wounds in the form of a suspension. When the gelatin microcarriers are exposed to the moist wound environment they aggregate and adhered to the wound bed with no formation of a gap between the dermal wound bed and the biomaterial. This is in accordance with previous work by the group [8] showing that the CultiSpher-S microcarriers can be injected and function as a dermal regeneration scaffold. Despite a more unorganized structure of the formed neoepidermis the immunohistochemical findings show that presence of microcarriers in the wounds does not disturb epidermal maturation in regard to keratinocyte differentiation and neoepidermal development. The barrier properties of the formed neoepidermis are of interest for future investigations.

Air-lifting of wounds was undertaken to investigate the process of stratification in wounds with microcarriers. Retaining sheets of epidermal cells in the air-liquid interphase, combined with inducing agents, leads to stratification and cornification of the epidermal layer within seven to ten days [22]. After air-lifting the wounds for the last seven days of 21 days of culture we observed similar stratification of the neoepidermis in wounds with microcarriers as in control wounds. This shows that the microcarriers do not interfere with the final step of maturation of the neoepidermis and that polarity of the keratinocytes is maintained within the scaffold.

We investigated whether a gelatin coating of the experimental wound bed would exert similar effects as the Cultispher-S gelatin microcarriers on re-epithelialization by adding 2% gelatin in solution to the wounds. The gelatin was left to settle in the wound area for 48 hours before the wound was submerged in culture medium. The results showed only partial re-epithelialization of all experimental wounds with a thick neoepidermal outgrowth. Keratinocytes suspended in semi-solid medium undergo terminal differentiation due to loss of contact to ECM [23,24] which would lead to halted proliferation and migration, and thereby inhibiting re-epithelialization. The appearance of single cells and clusters of cells in the wound bed of wounds cultured with gelatin solution could be explained by the induction of incomplete differentiation of those cells. These findings show that the scaffolding function of the gelatin microcarriers is important for successful re-epithelialization and that it can be achieved by aggregated CultiSpher-S microcarriers.

Disruption of the skin barrier by external wounding leads to vast cellular and biochemical actions to restore barrier integrity. A main event of the proliferative phase of wound healing is the activation of keratinocytes at the wound edge, aimed to migrate and re-epithelialize the wounded area. In the case of extensive damage, or stagnation into a chronic wound, the possibility for successful migration of wound edge keratinocytes is limited due to an overall imbalance of the wound healing microenvironment attributed to ongoing inflammation, lack of substrate and inappropriate signaling cues [25]. Chronic wounds are a serious challenge in wound management so further investigations on the suitability of the CultiSpher-S as scaffolding in a chronic wound model would be of interest.

The present study shows that Cultispher-S microcarriers can function as tissue guiding scaffold for keratinocytes for re-epithelialization of *in vitro* cutaneous wounds without additional cell transplantation. The *in vitro* model is based on viable human skin with cells present relevant for re-epithelialization giving strong indications for the *in vivo* situation. The microcarriers facilitated the formation of a thick epidermis while supporting maturation of the neoepidermis. Previous work shows the microcarriers to be suitable as a guiding scaffold for dermal regeneration and together with our findings we conclude that the CultiSpher-S microcarriers can be beneficial as provisional matrix for cutaneous wound healing. A microcarrier suspension is easy to store and handle, administration is direct and the microcarriers in suspension can fill in wounds of various shapes and depths which infers that the CultiSpher-S microcarriers show an advantage over static sheet shaped scaffolding.

## Supporting Information

S1 Datatable Thickness measurement dataset. Description of the measurement procedure in Fiji and the obtained measurement values for wound sections analyzed in the study.
(PDF)

S1 Fig Representative image of junction between microcarriers and the dermal wound bed. Hematoxylin-eosin staining of wound cultured with microcarriers for 21 days. The organization of collagen fibers is longitudinal adjacent to the microcarriers and no adverse effects can be observed. Asterisk (*) indicates blood vessels in the dermal wound bed. Scale bar = 200 μm.
(TIF)

S2 Fig Representative images of wounds cultured with 2% gelatin solution for 21 days. (A and B) Hematoxylin-eosin staining of section of wound cultured with 2% gelatin. Note single cells and cell clusters indicated by asterisk (*) in the wound bed in magnification (A). (C) Immunohistochemical staining against pancytokeratin (red) and nuclear staining with 4, 6-diamidino-2-phenylindole (DAPI). Arrow indicates neoepidermis, arrowheads indicate wound edges. Scale bars = 200 μm for (A), 1000 μm for (B) and (C).
(TIF)

S1 Imageset Collection of staining raw data images quantified in S1 Datatable. (ZIP)
