# Supplementary material for: Biodegradable Gelatin Microcarriers Facilitate Re-Epithelialization of Human Cutaneous Wounds - An In Vitro Study in Human Skin
Source: PLoS One. 2015 Jun 10;10(6):e0128093. doi: 10.1371/journal.pone.0128093 (PMC4464648; doi:10.1371/journal.pone.0128093)

ImageJ/Fiji procedure:

Set scale

Threshold select

ROI manager-> add

line along base -> measure (length)

bounding box -> add

threshold AND bounding box ->measure (area)

1 µm =	0.948 pixels

Measurements

	**Sample**	*Area of stain*	*Length (px)*	**Area/length (thickness, µm)**
**Control**	131009 c d21 1.1	119654.525	2350.051	48.26809703
	131009 c d21 1.2	154662.714	1975.066	74.23562193
	131009 c d21 1.3	99259.6	2381.207	39.51697639
	121010 c d21 1.1	103777.217	2671.194	38.85049794
	121010 c d21 1.2	90109.7558	2338.405	38.53470883
	121010 c d21 2.1	116929.151	2924.004	39.98939502
	121010 c d21 2.2	136232.842	2885.162	47.21843765
	121010 c d21 2.3	131412.567	2986.219	44.00633945
	121010 c d21 3.1	94266.855	1963.819	48.00180414
	121010 c d21 3.2	101511.732	1943.288	52.23710124
	121010 c d21 3.3	104686.304	1993.194	52.52188397
**Microcarriers**	131009 mc d21 4.1	181928.644	1916.093	90.01042982
	131009 mc d21 4.2	300181.15	2286.626	124.4504918
	131009 mc d21 4.3	115129.119	1591.18	68.59211705
	131009 mc d21 5.1	159559.766	2215.412	68.27743922
	131009 mc d21 5.2	141587.219	2090.789	64.19810111
	131009 mc d21 5.3	181069.629	2274.386	75.47268067
	131009 mc d21 6.1	519292.225	3072.202	160.2397985
	131009 mc d21 6.2	205614.974	2702.781	72.11941898
	131009 mc d21 6.3	311609.829	2652.709	111.3601672
	121010 mc d21 1.1	330924.309	3104.919	106.5806577
	121010 mc d21 1.2	361614.058	3072.876	117.6793525
	121010 mc d21 1.3	377808.489	3178.719	118.8555796
	121010 mc d21 3.1	573495.834	2331.056	246.0240483
	121010 mc d21 3.2	518020.394	2176.636	237.9912829
	121010 mc d21 3.3	670646.843	2594.848	258.4532285

Results

	MC	Ctrl
	90.01043	48.2681
	124.4505	74.23562
	68.59212	39.51698
	68.27744	38.8505
	64.1981	38.53471
	75.47268	39.9894
	160.2398	47.21844
	72.11942	44.00634
	111.3602	48.0018
	106.5807	52.2371
	117.6794	52.52188
	118.8556	
	246.024	
	237.9913	
	258.4532	
Stdev:	*67.3558451*	*10.25103553*
TTEST:	p =	0.000194
Avg thickness (um):	128.0203	47.58008
Comparative %:	269%	37%

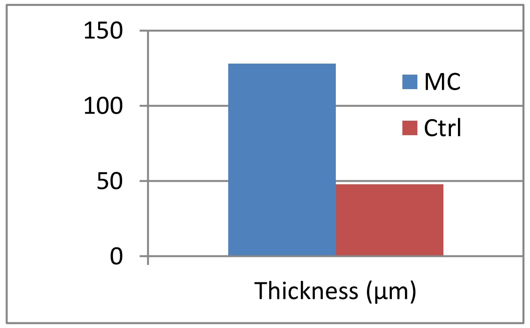

Supplement: S1 Datatable — Description of the measurement procedure in Fiji and the obtained measurement values for wound sections analyzed in the study. (PDF) [file pone.0128093.s001.pdf]
