# Supplementary figures and images for: Biodegradable Gelatin Microcarriers Facilitate Re-Epithelialization of Human Cutaneous Wounds - An In Vitro Study in Human Skin
Source: PLoS One. 2015 Jun 10;10(6):e0128093. doi: 10.1371/journal.pone.0128093 (PMC4464648; doi:10.1371/journal.pone.0128093)

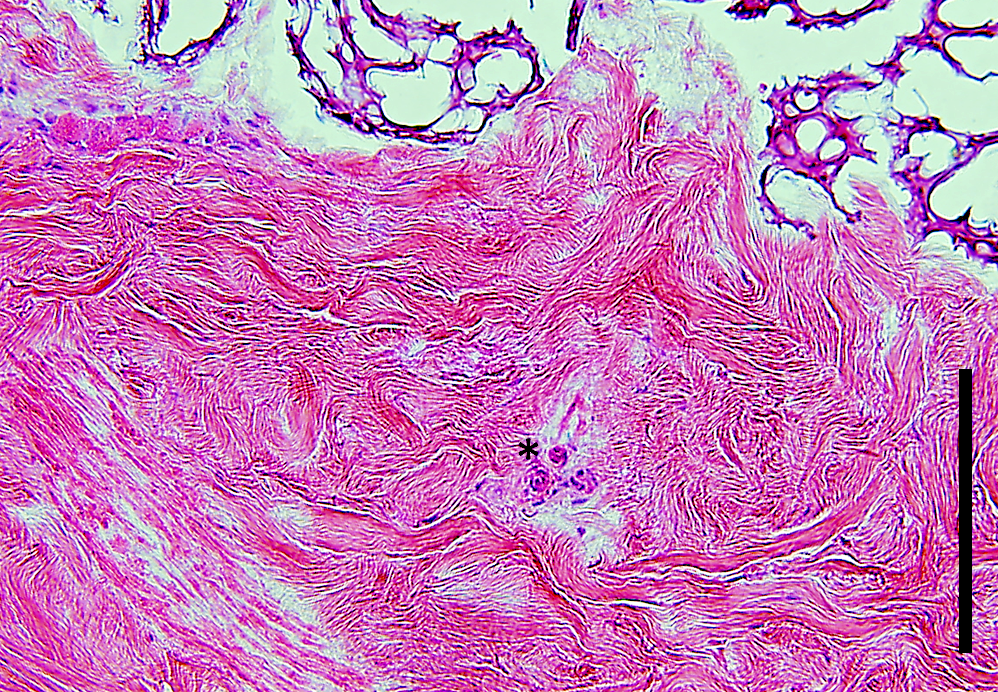

Supplement: S1 Fig — Hematoxylin-eosin staining of wound cultured with microcarriers for 21 days. The organization of collagen fibers is longitudinal adjacent to the microcarriers and no adverse effects can be observed. Asterisk (*) indicates blood vessels in the dermal wound bed. Scale bar = 200 μm. (TIF) [file pone.0128093.s002.tif]

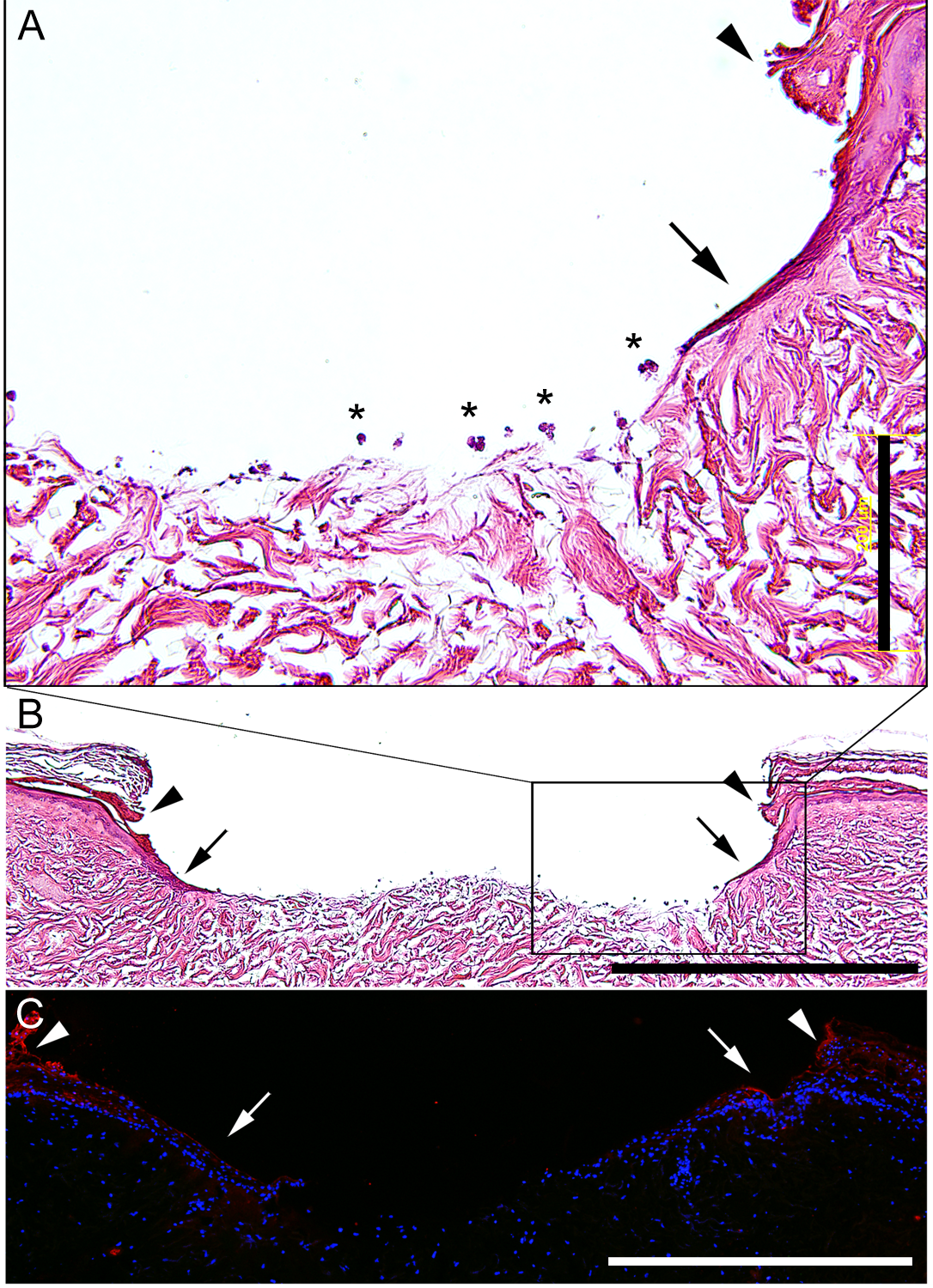

Supplement: S2 Fig — (A and B) Hematoxylin-eosin staining of section of wound cultured with 2% gelatin. Note single cells and cell clusters indicated by asterisk (*) in the wound bed in magnification (A). (C) Immunohistochemical staining against pancytokeratin (red) and nuclear staining with 4, 6-diamidino-2-phenylindole (DAPI). Arrow indicates neoepidermis, arrowheads indicate wound edges. Scale bars = 200 μm for (A), 1000 μm for (B) and (C). (TIF) [file pone.0128093.s003.tif]

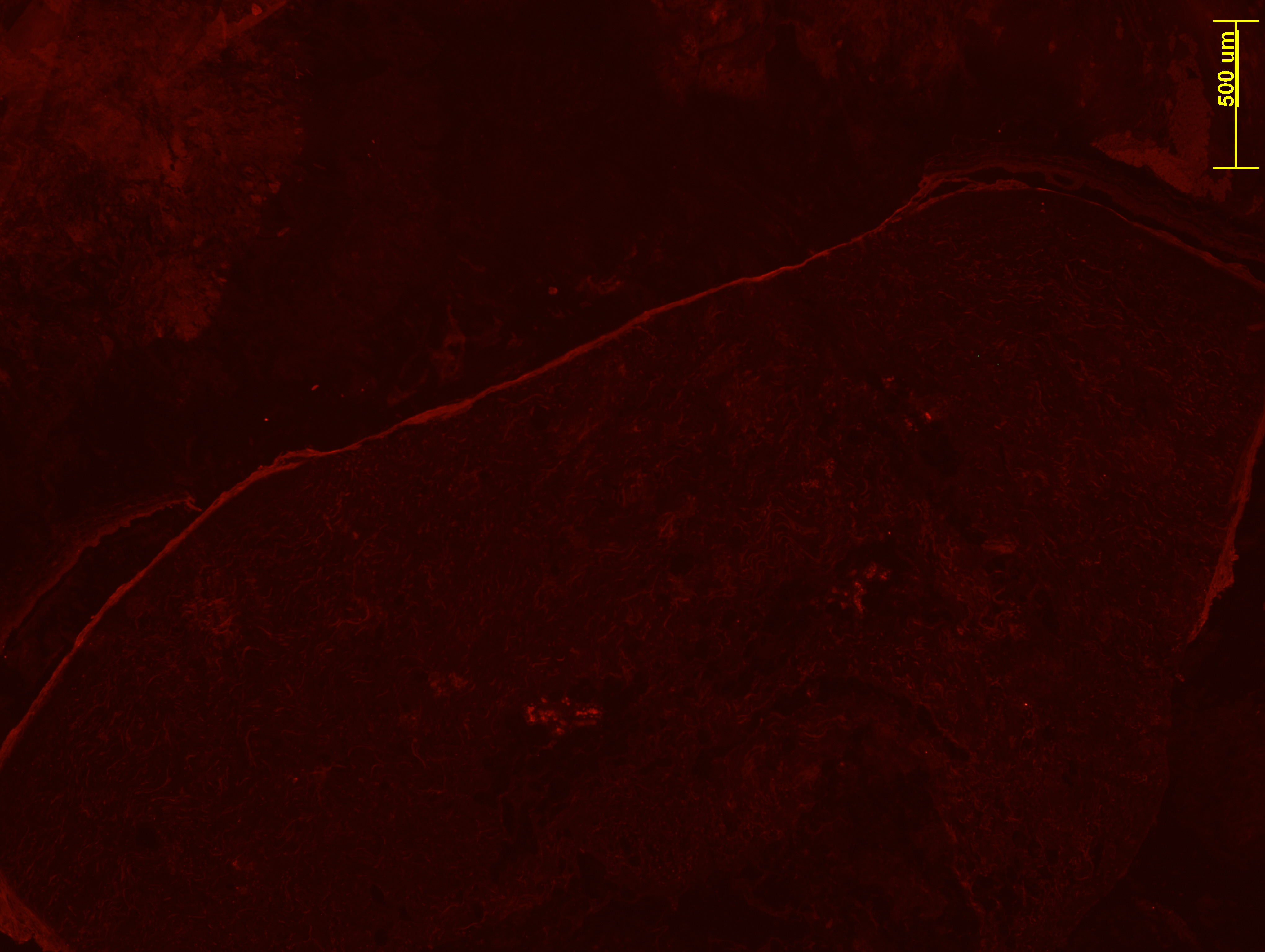

Supplement: S1 Imageset — (ZIP) [file pone.0128093.s004.zip › Immunos/Controls/121010 mc d21 1.1 control 4x.jpg]

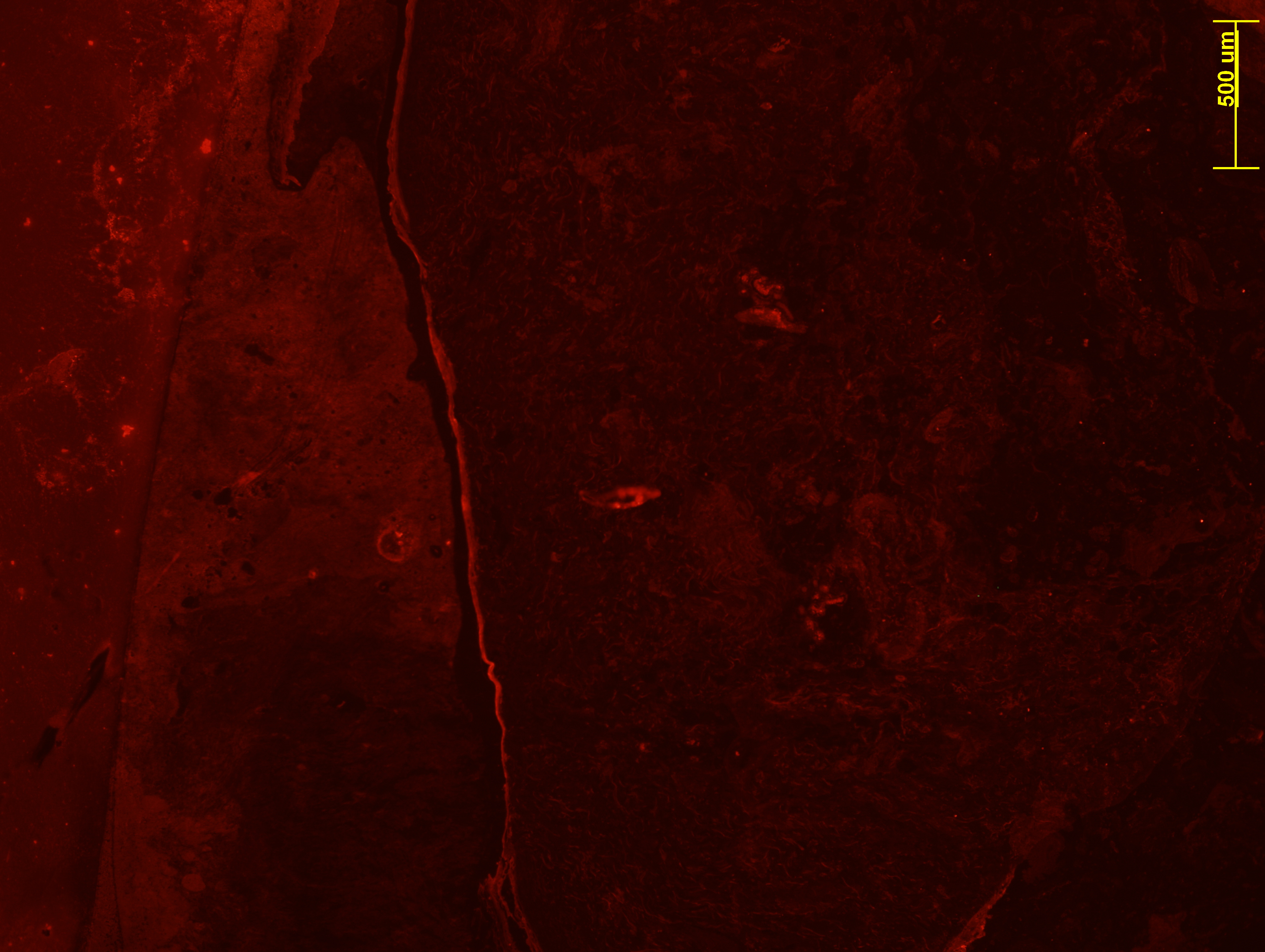

Supplement: S1 Imageset — (ZIP) [file pone.0128093.s004.zip › Immunos/Controls/121010 mc d21 1.2 control 4x.jpg]

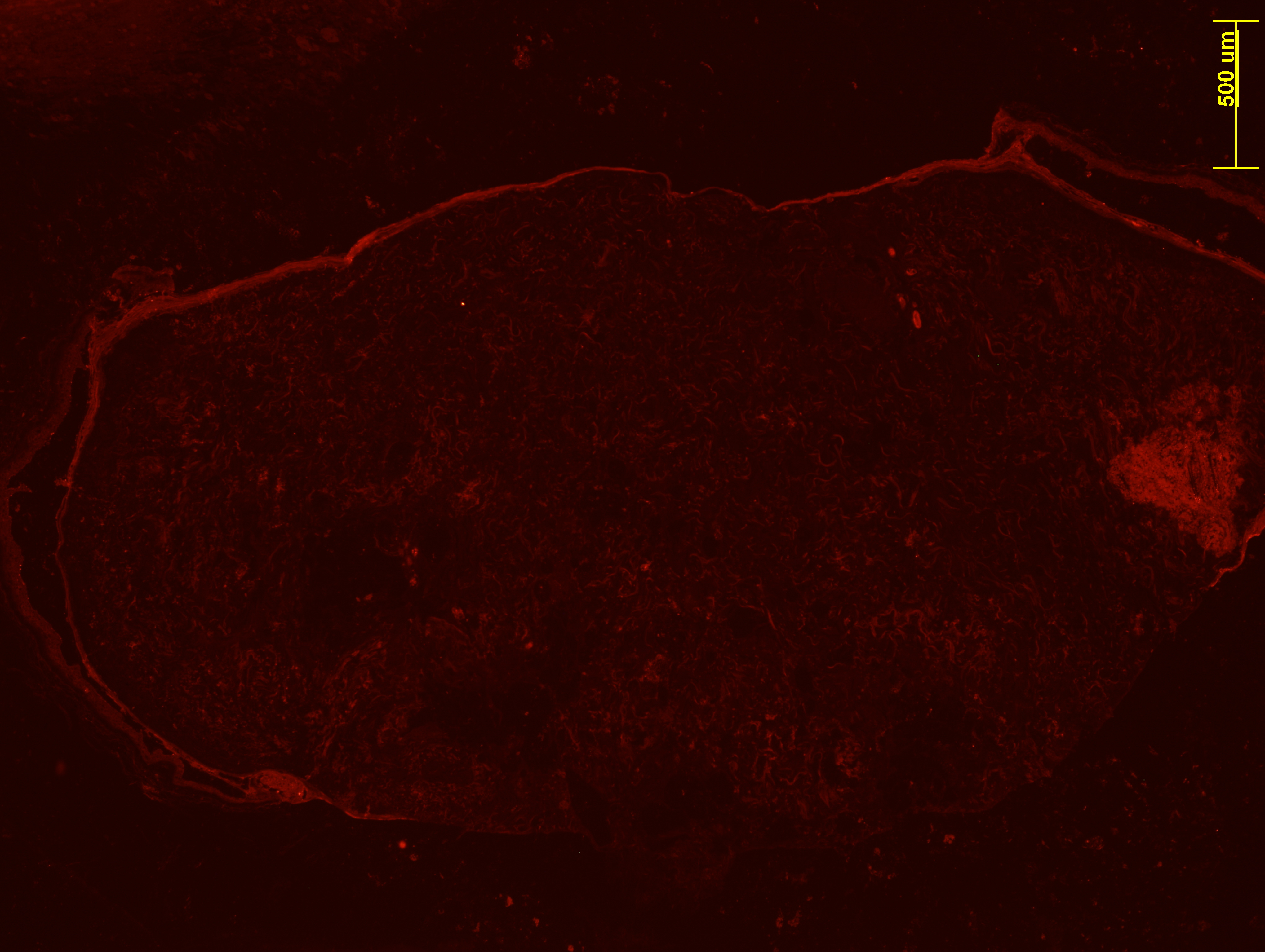

Supplement: S1 Imageset — (ZIP) [file pone.0128093.s004.zip › Immunos/Controls/121010 mc d21 2.1 control 4x.jpg]

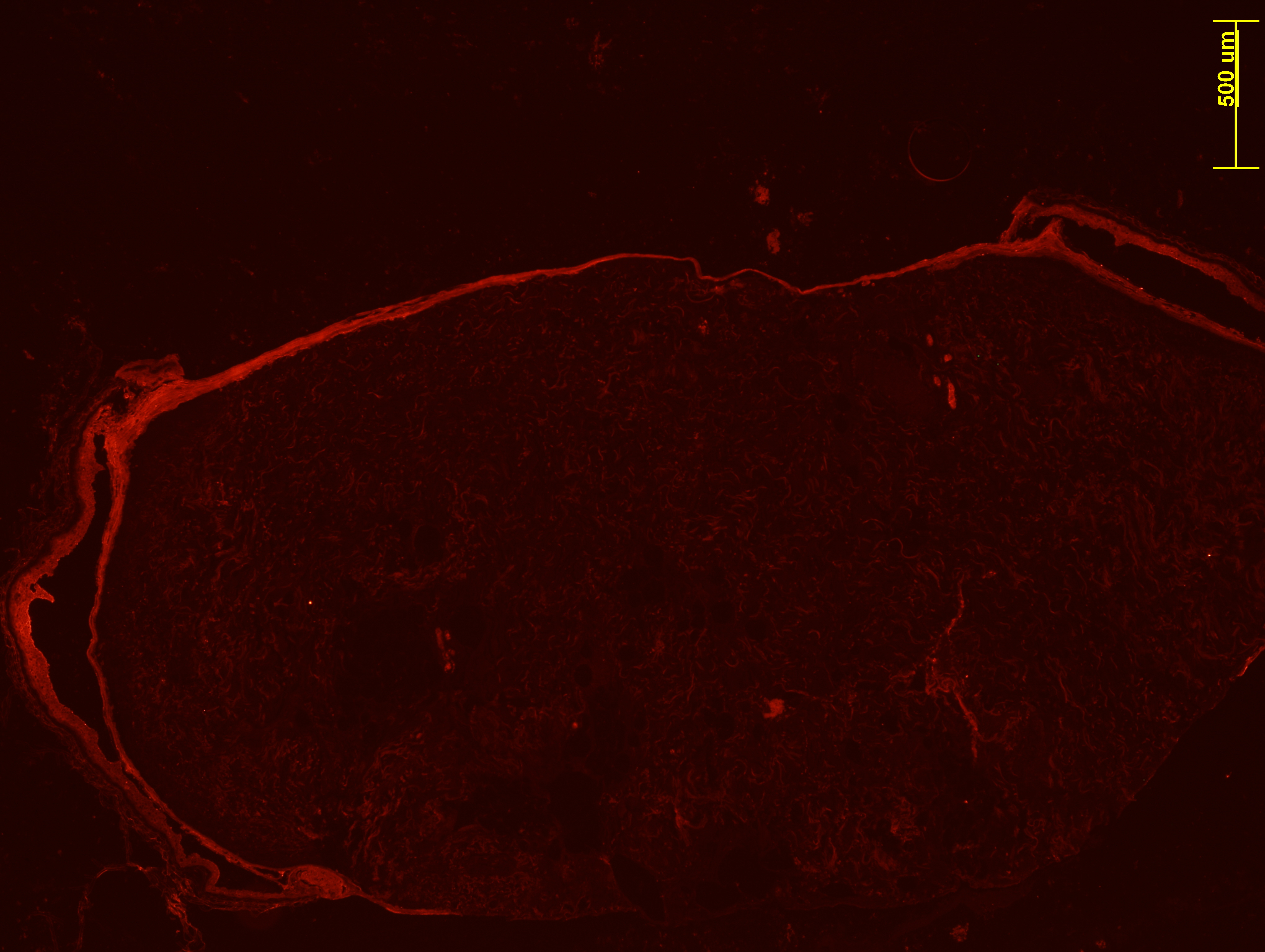

Supplement: S1 Imageset — (ZIP) [file pone.0128093.s004.zip › Immunos/Controls/121010 mc d21 2.2 control 4x.jpg]

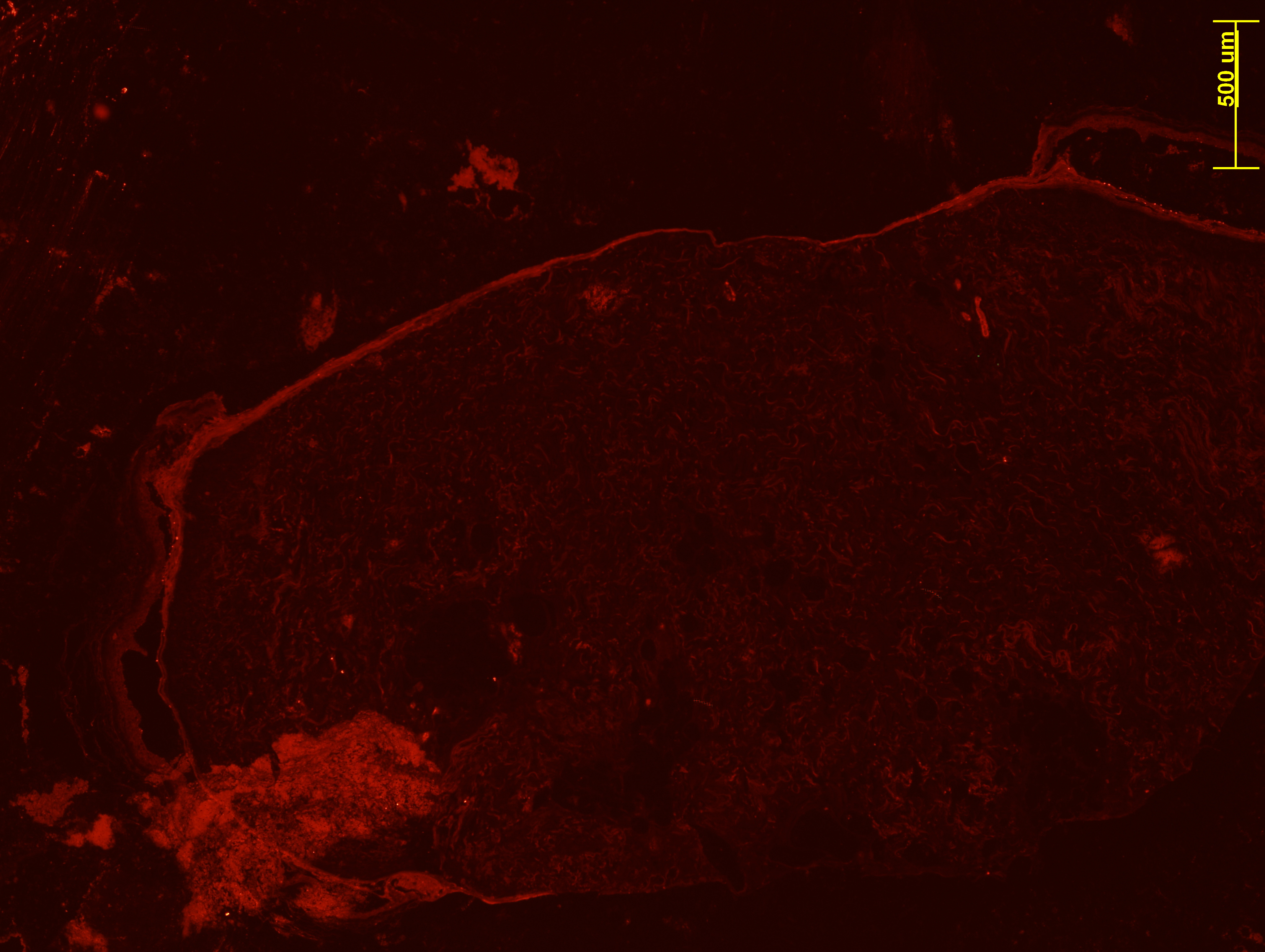

Supplement: S1 Imageset — (ZIP) [file pone.0128093.s004.zip › Immunos/Controls/121010 mc d21 2.3 control 4x.jpg]

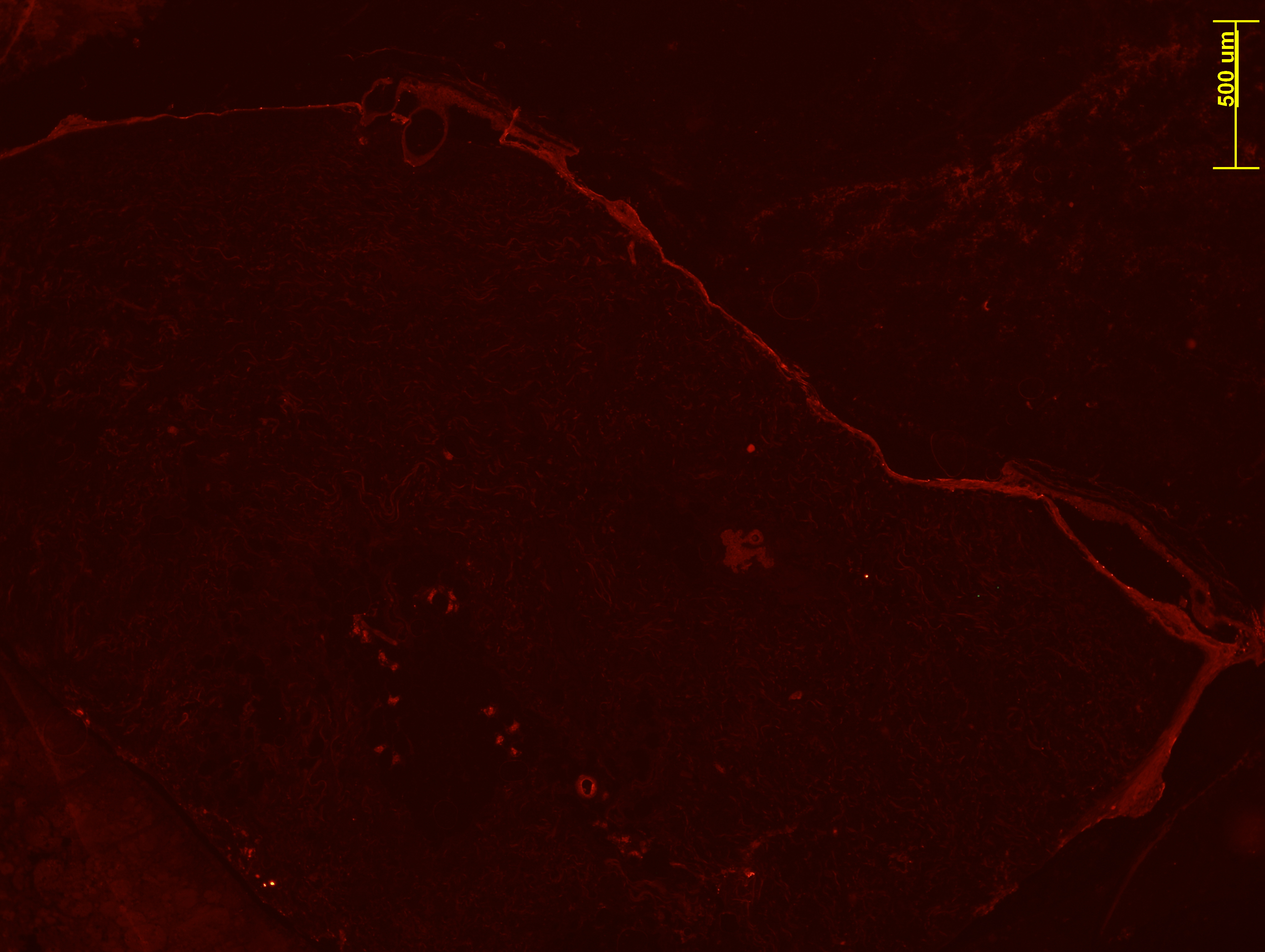

Supplement: S1 Imageset — (ZIP) [file pone.0128093.s004.zip › Immunos/Controls/121010 mc d21 3.1 control 4x.jpg]

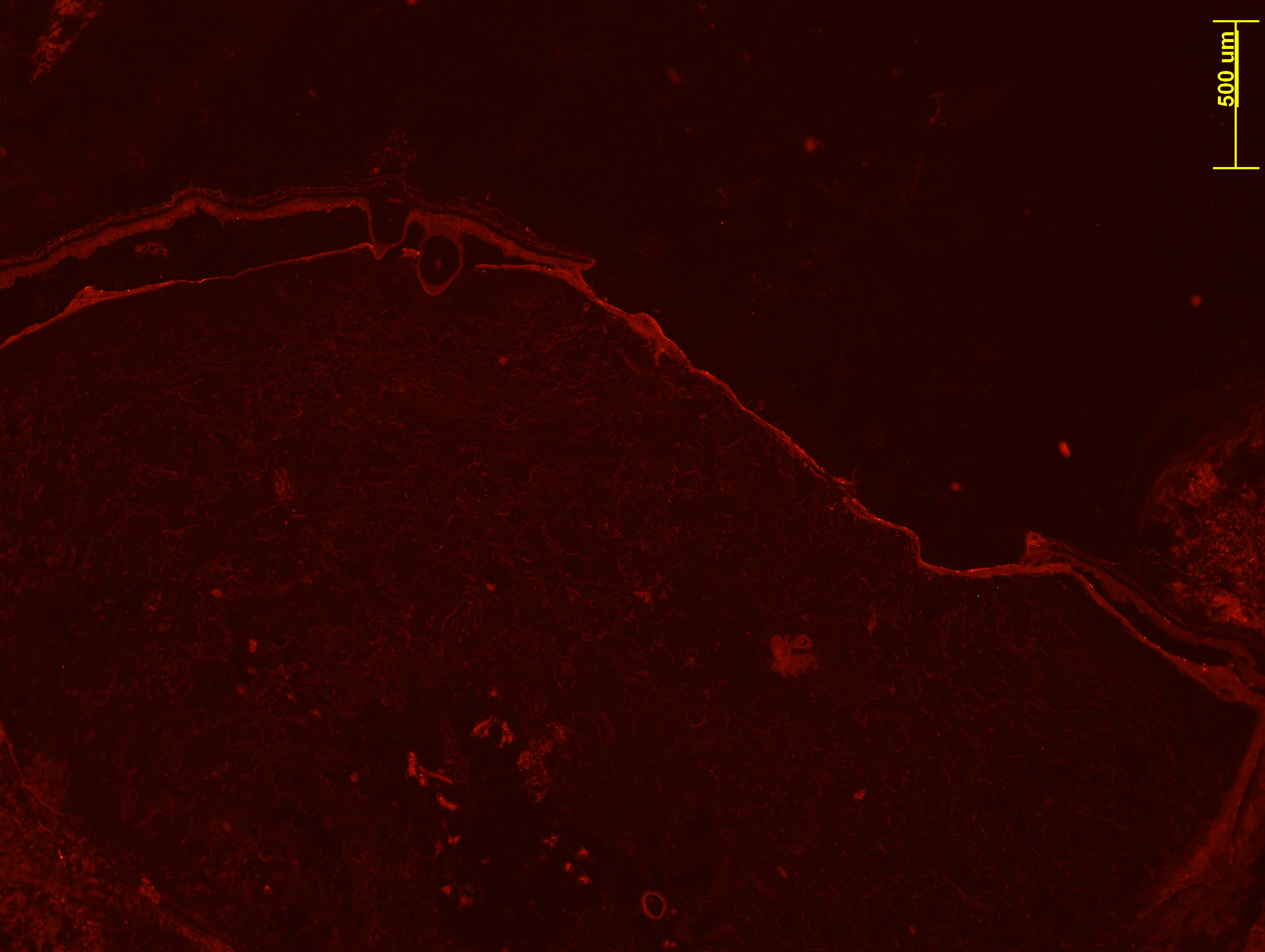

Supplement: S1 Imageset — (ZIP) [file pone.0128093.s004.zip › Immunos/Controls/121010 mc d21 3.2 control 4x.jpg]

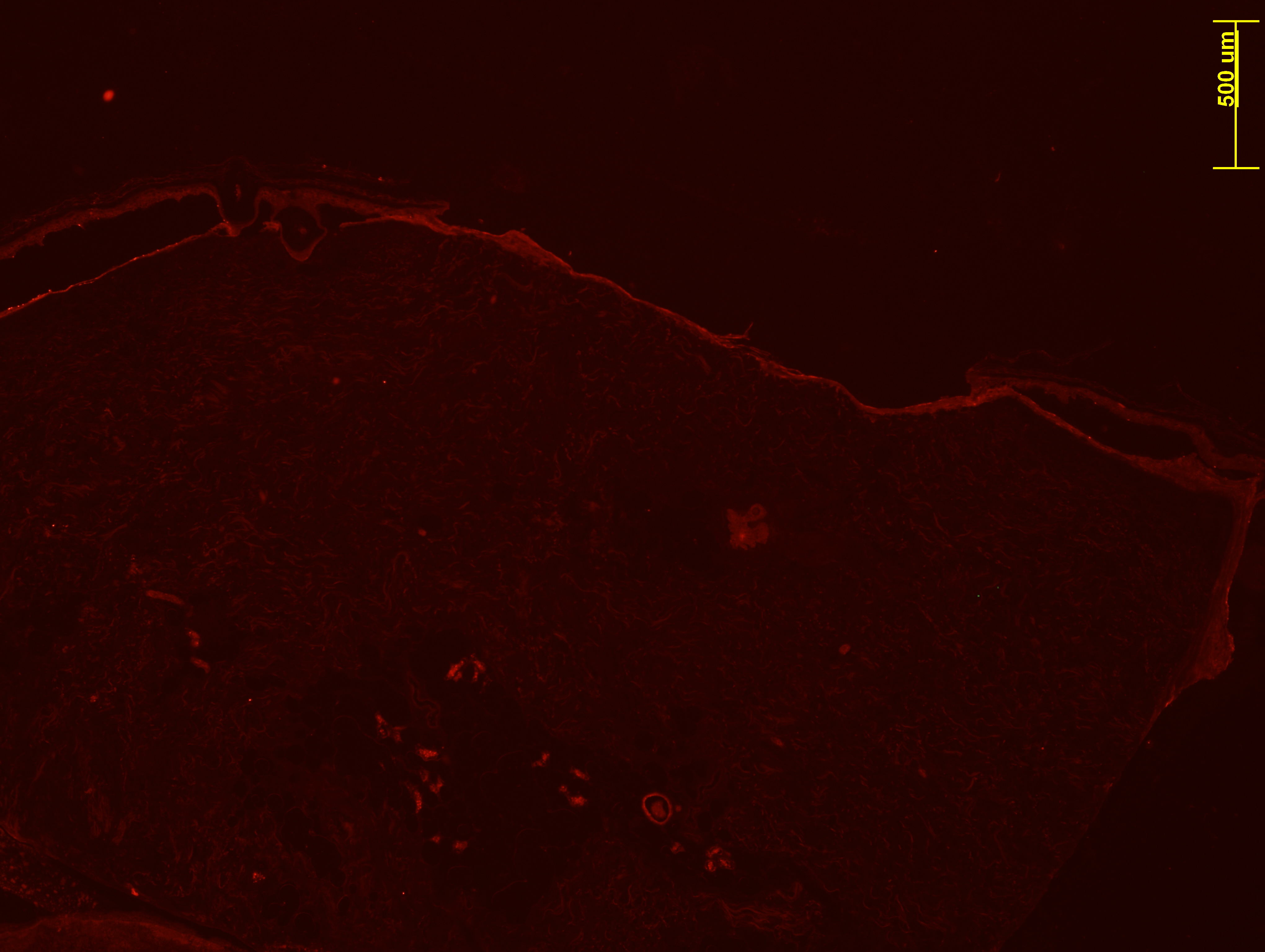

Supplement: S1 Imageset — (ZIP) [file pone.0128093.s004.zip › Immunos/Controls/121010 mc d21 3.3 control 4x.jpg]

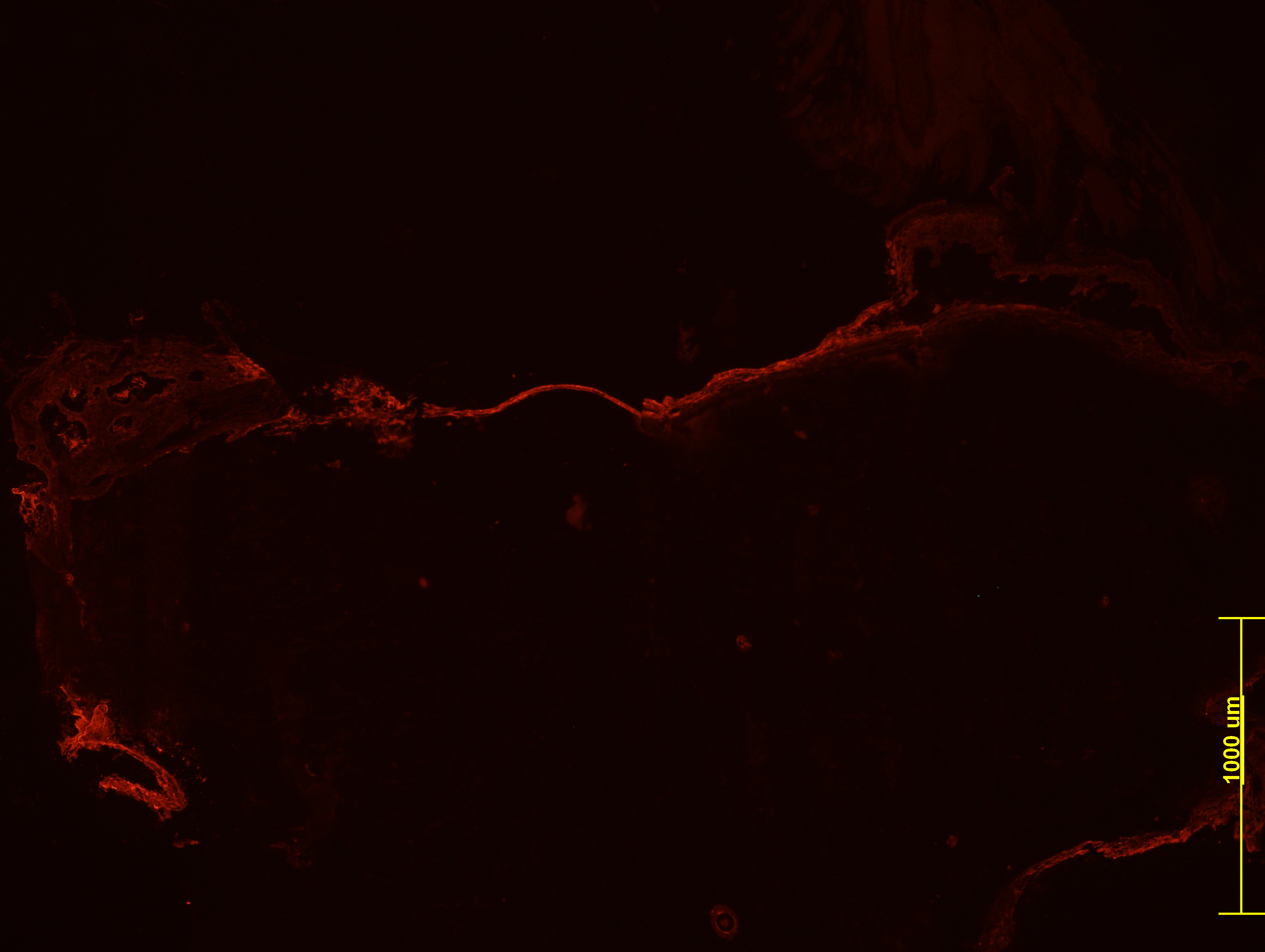

Supplement: S1 Imageset — (ZIP) [file pone.0128093.s004.zip › Immunos/Controls/131009 mc d21 1.1 control 4x.jpg]

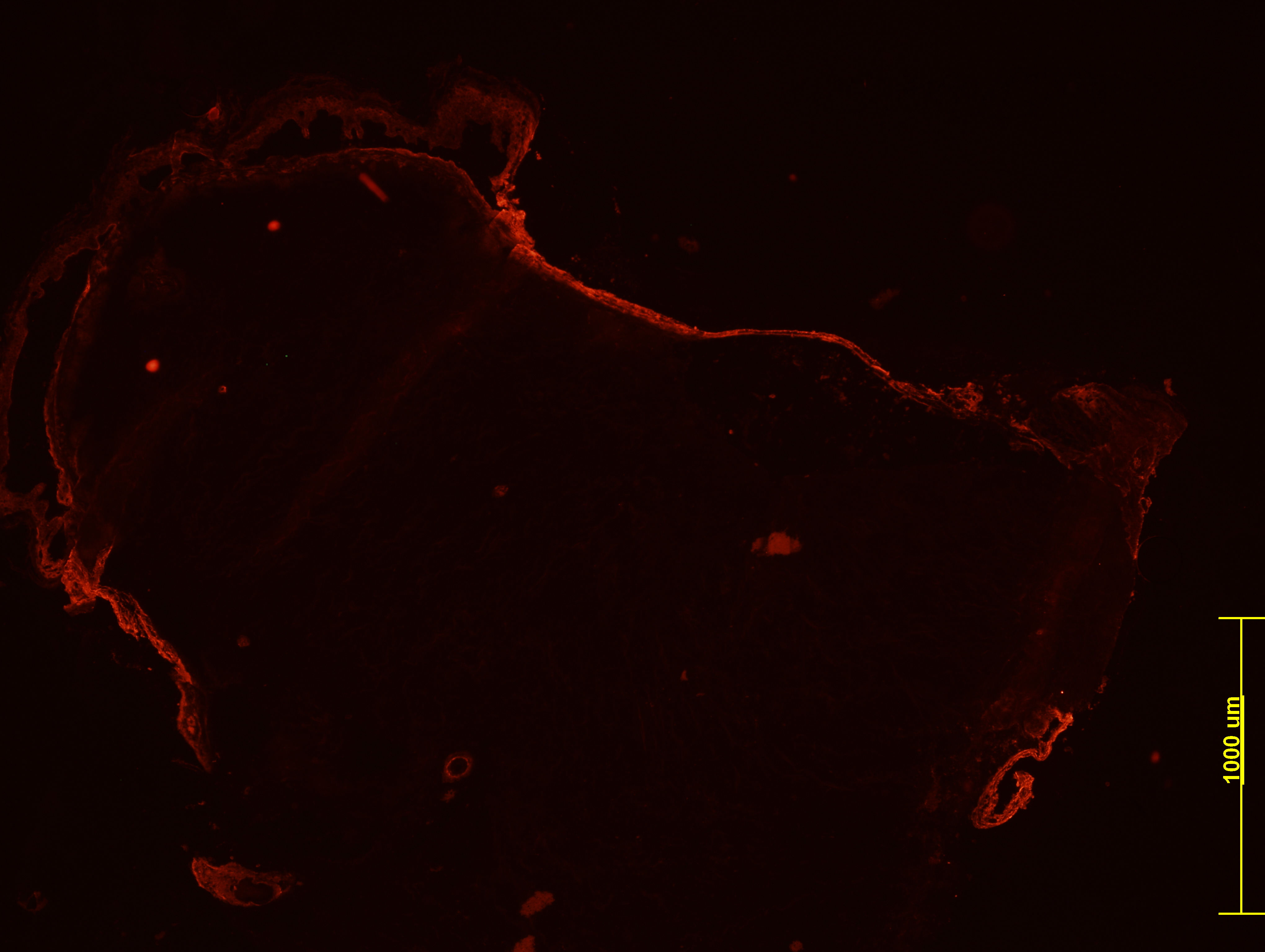

Supplement: S1 Imageset — (ZIP) [file pone.0128093.s004.zip › Immunos/Controls/131009 mc d21 1.2 control 4x.jpg]

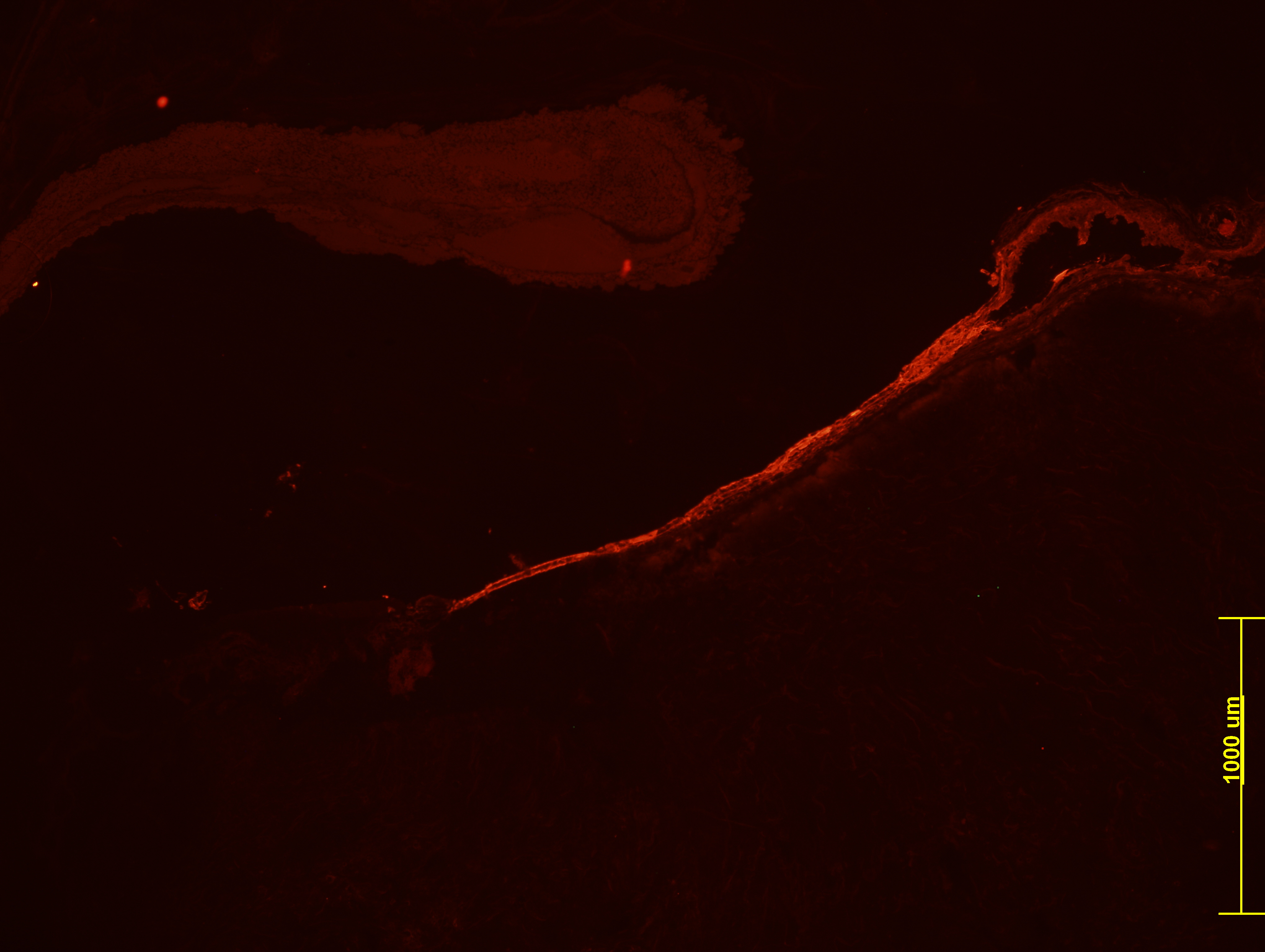

Supplement: S1 Imageset — (ZIP) [file pone.0128093.s004.zip › Immunos/Controls/131009 mc d21 1.3 control 4x.jpg]

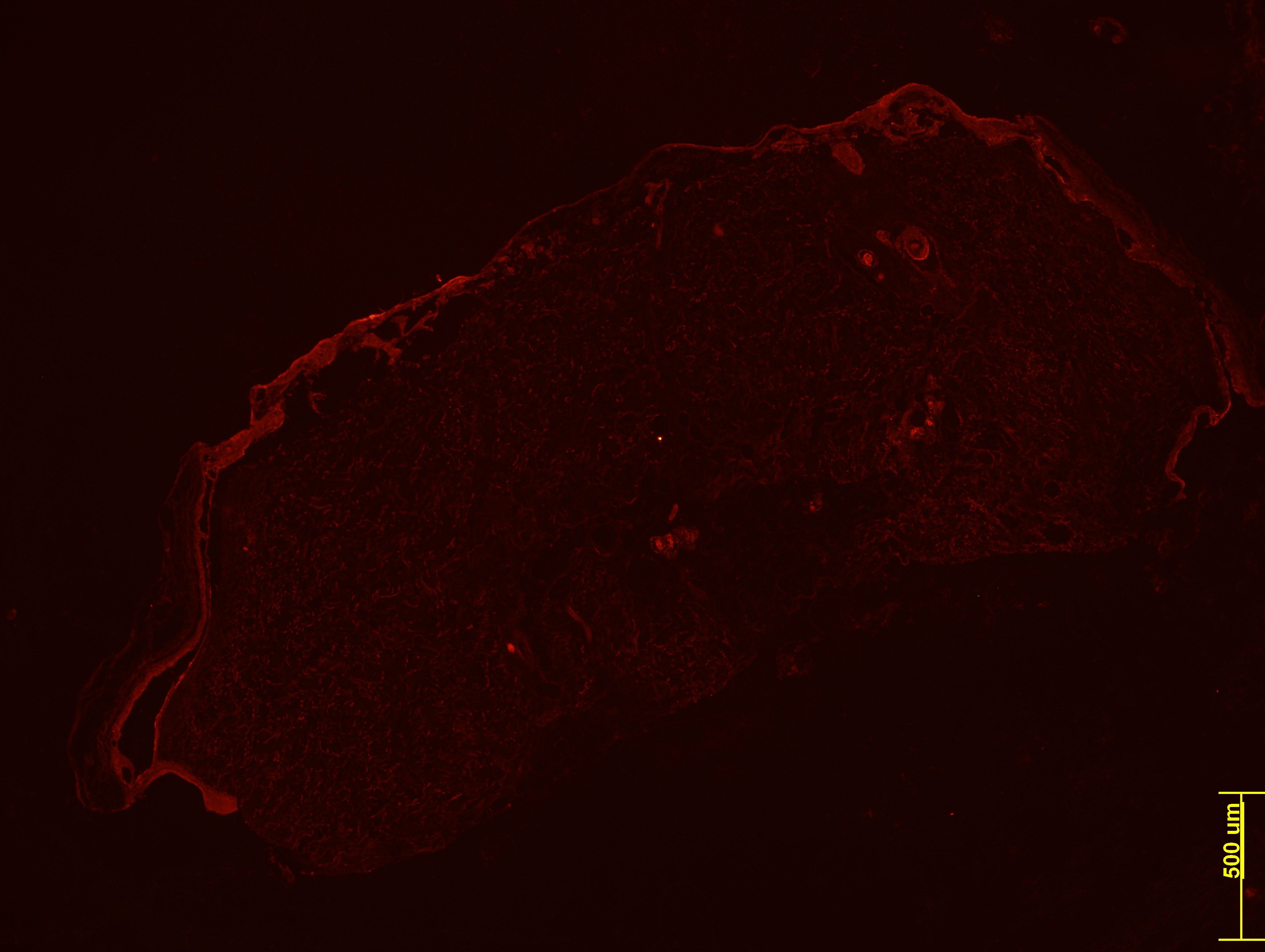

Supplement: S1 Imageset — (ZIP) [file pone.0128093.s004.zip › Immunos/MC/121010 mc 21d 1.1 4x.jpg]

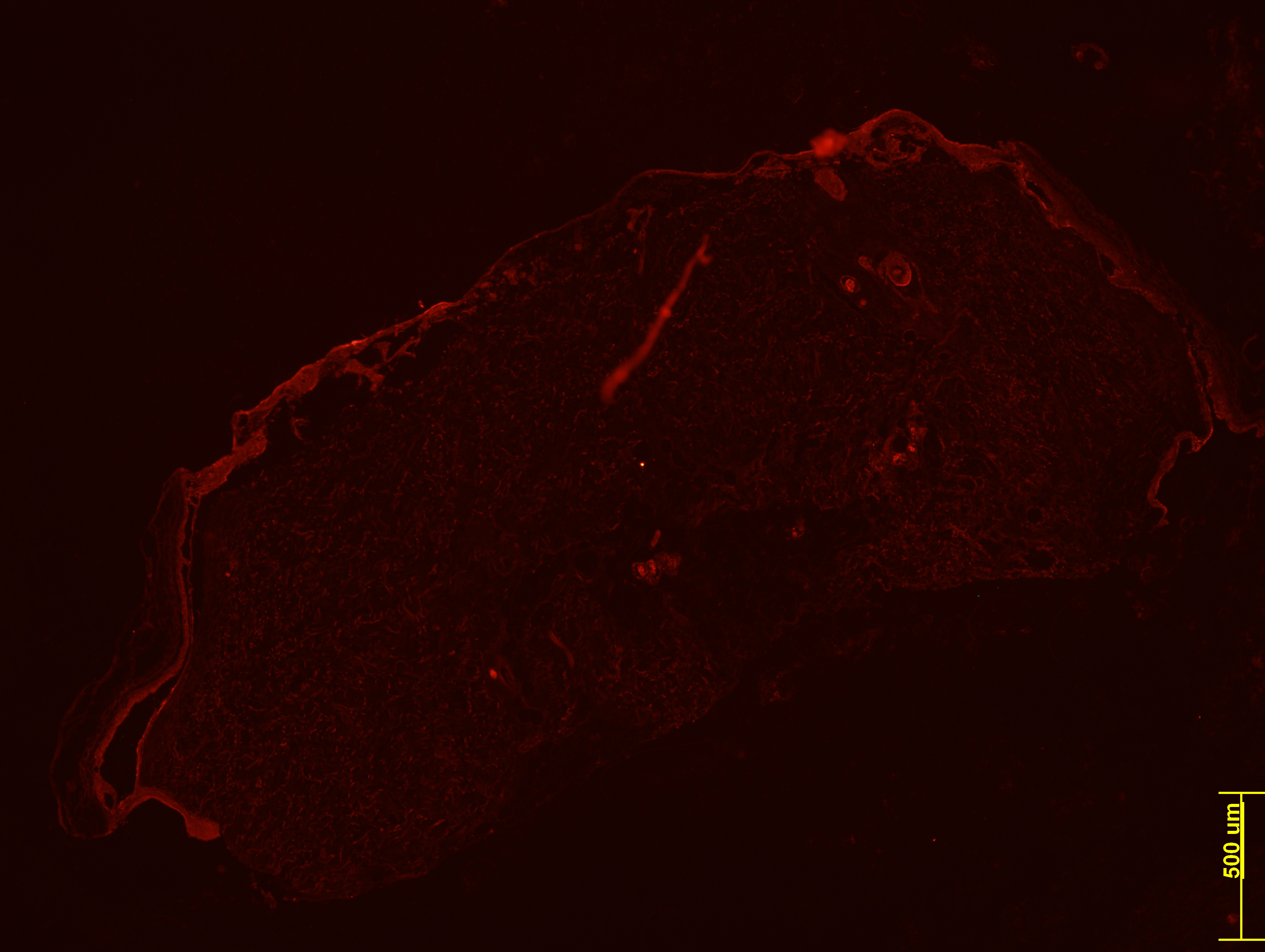

Supplement: S1 Imageset — (ZIP) [file pone.0128093.s004.zip › Immunos/MC/121010 mc 21d 1.2 4x.jpg]

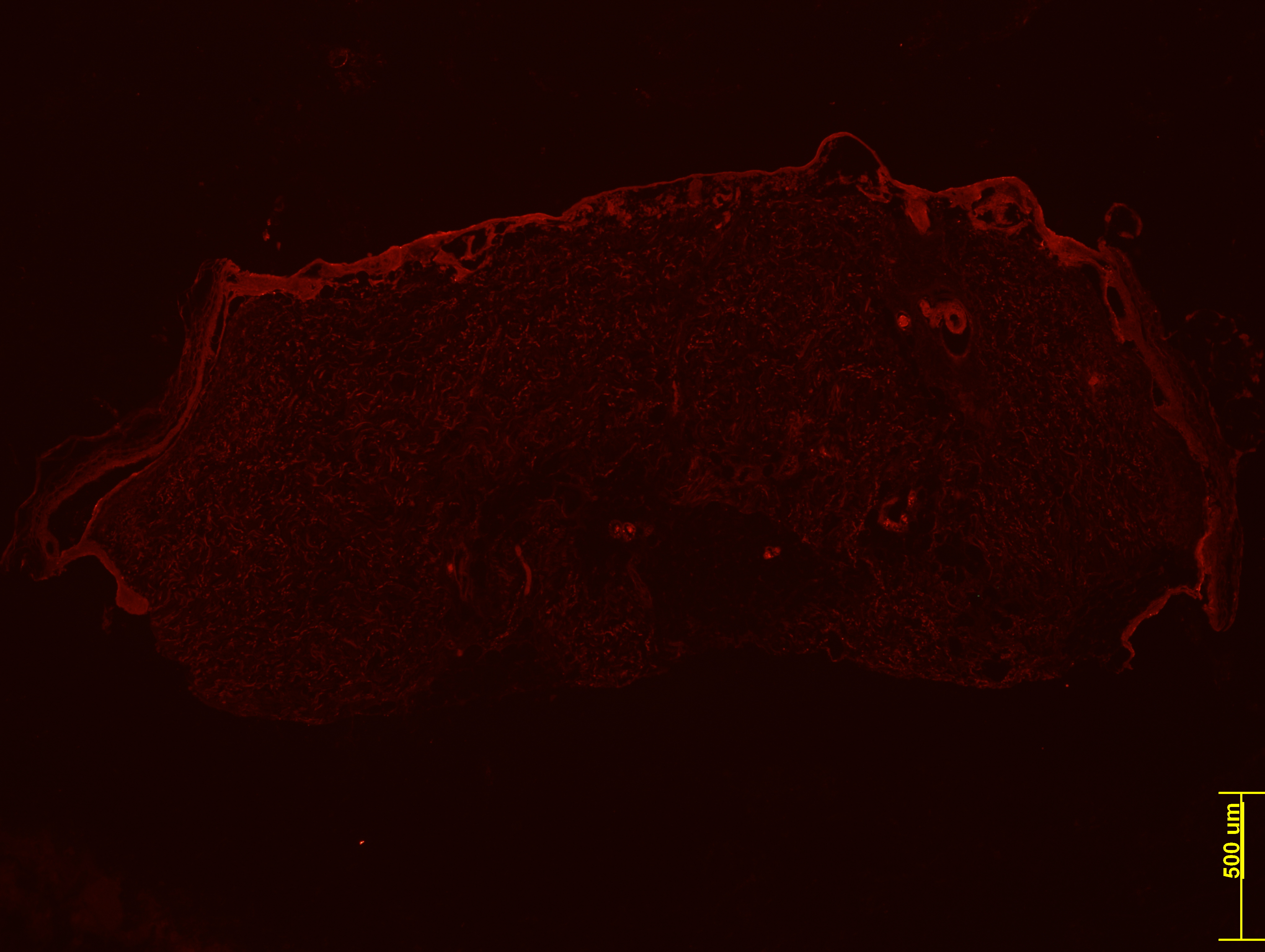

Supplement: S1 Imageset — (ZIP) [file pone.0128093.s004.zip › Immunos/MC/121010 mc 21d 1.3 4x.jpg]

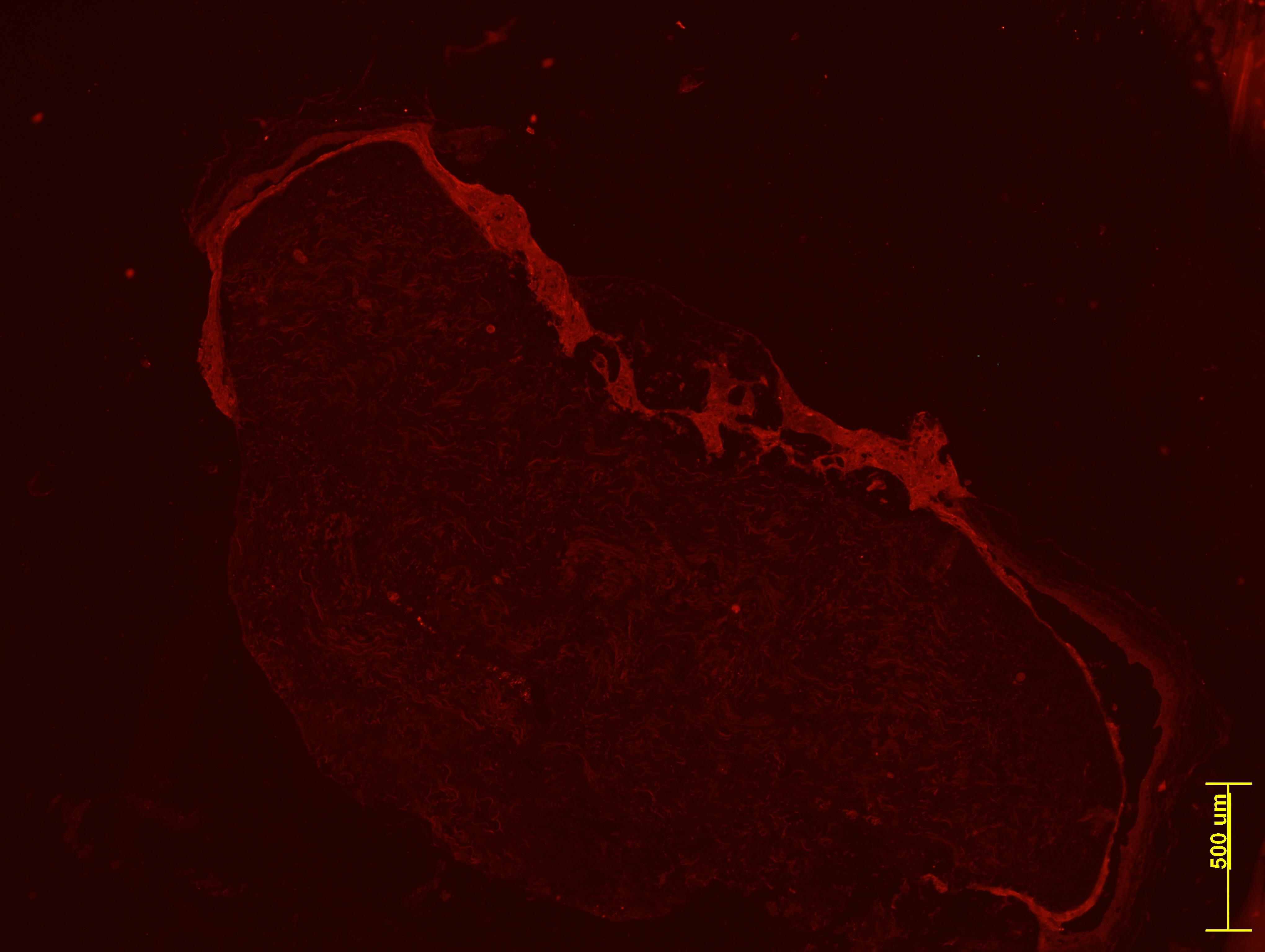

Supplement: S1 Imageset — (ZIP) [file pone.0128093.s004.zip › Immunos/MC/121010 mc d21 3.1 4x.jpg]

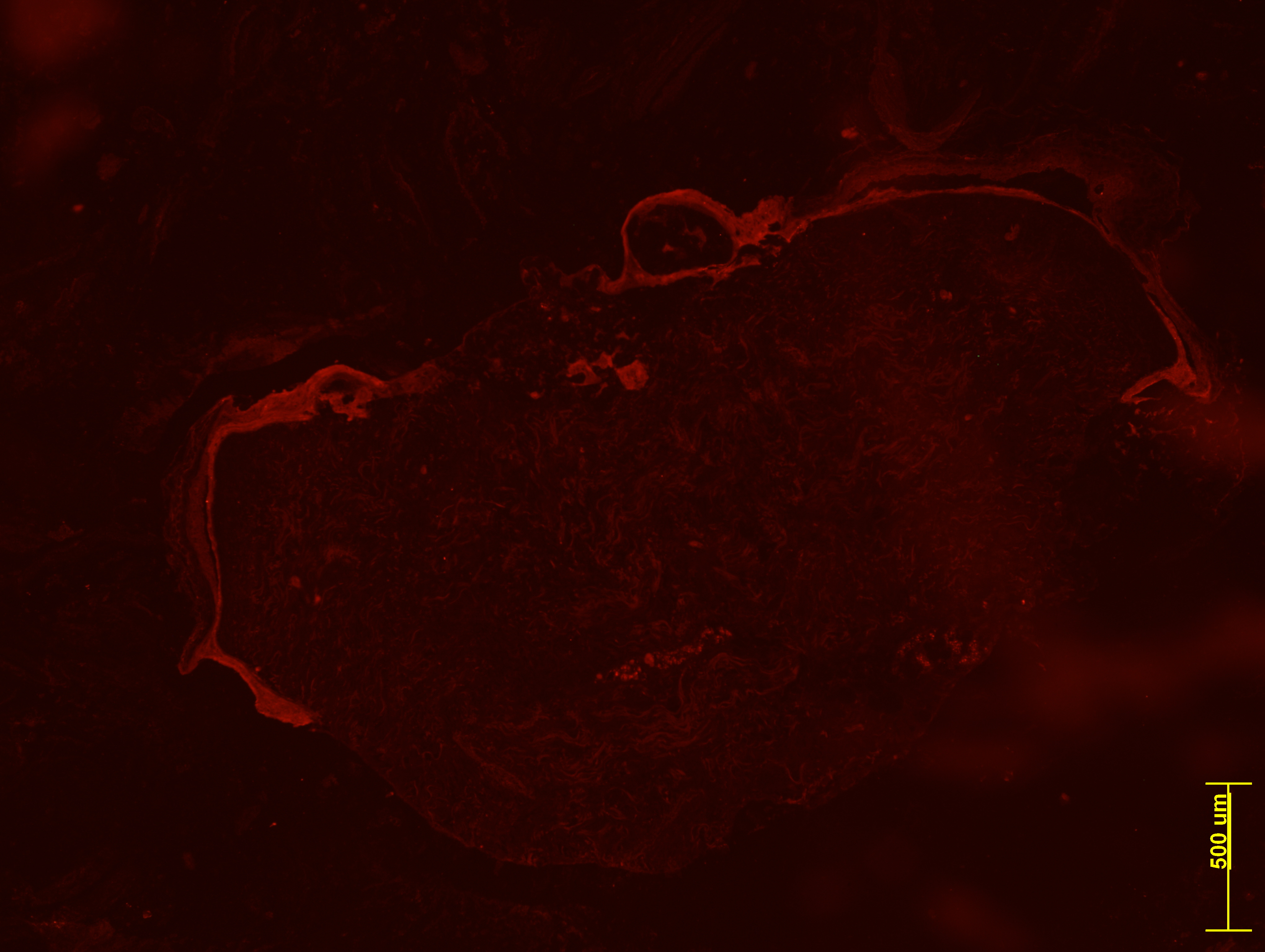

Supplement: S1 Imageset — (ZIP) [file pone.0128093.s004.zip › Immunos/MC/121010 mc d21 3.2 4x.jpg]

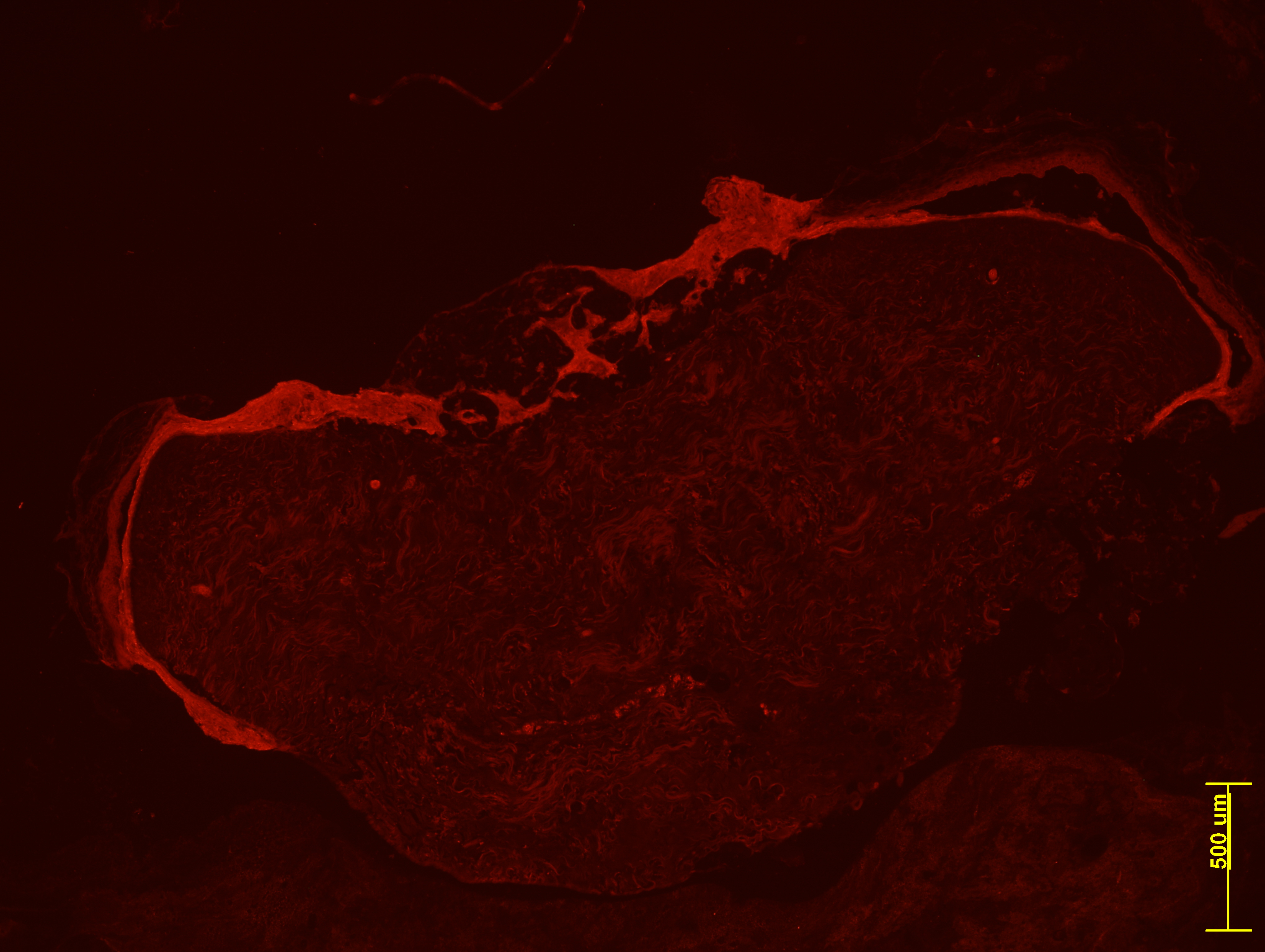

Supplement: S1 Imageset — (ZIP) [file pone.0128093.s004.zip › Immunos/MC/121010 mc d21 3.3 4x.jpg]

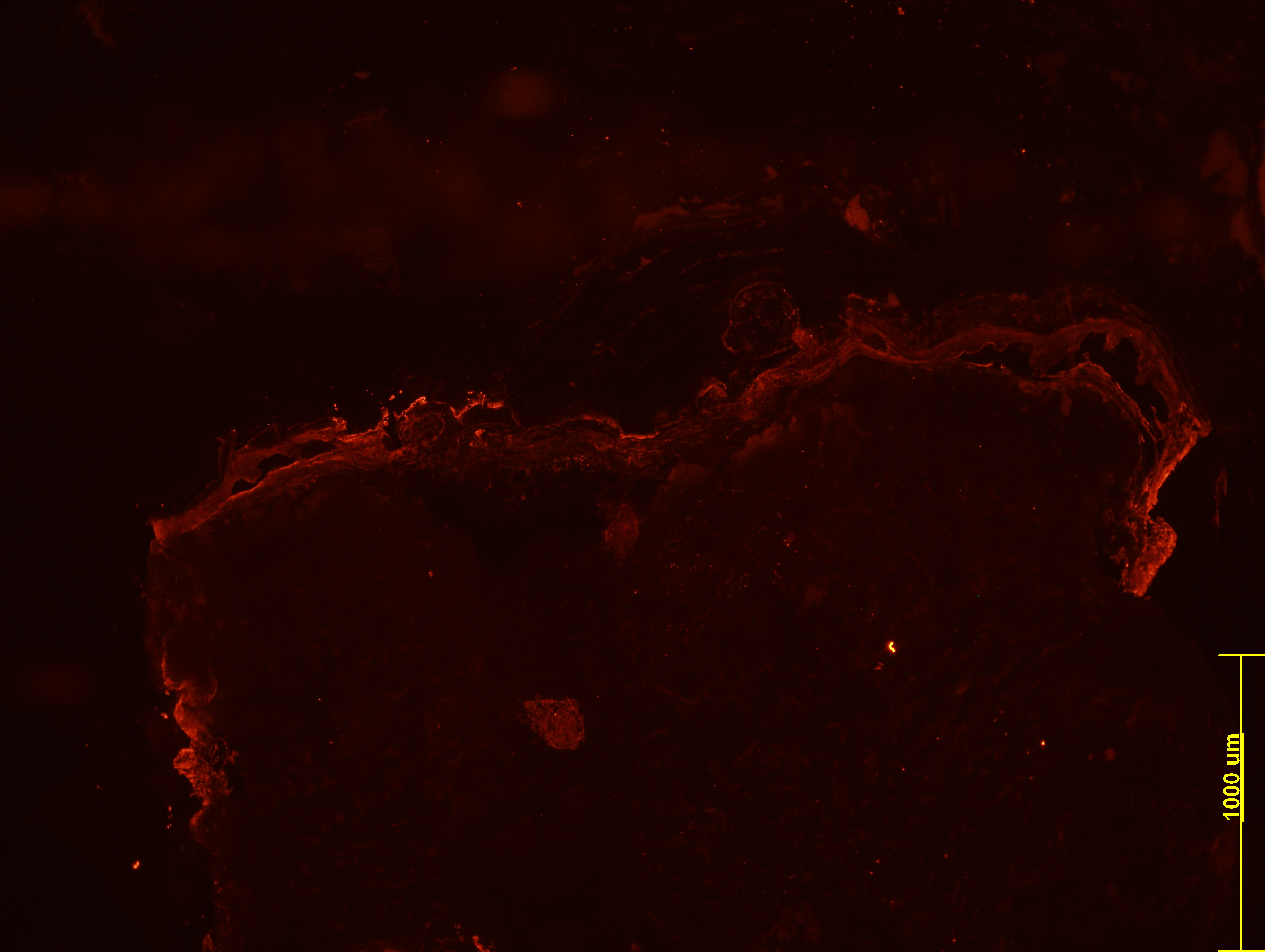

Supplement: S1 Imageset — (ZIP) [file pone.0128093.s004.zip › Immunos/MC/131009 mc d21 4.1 4x.jpg]

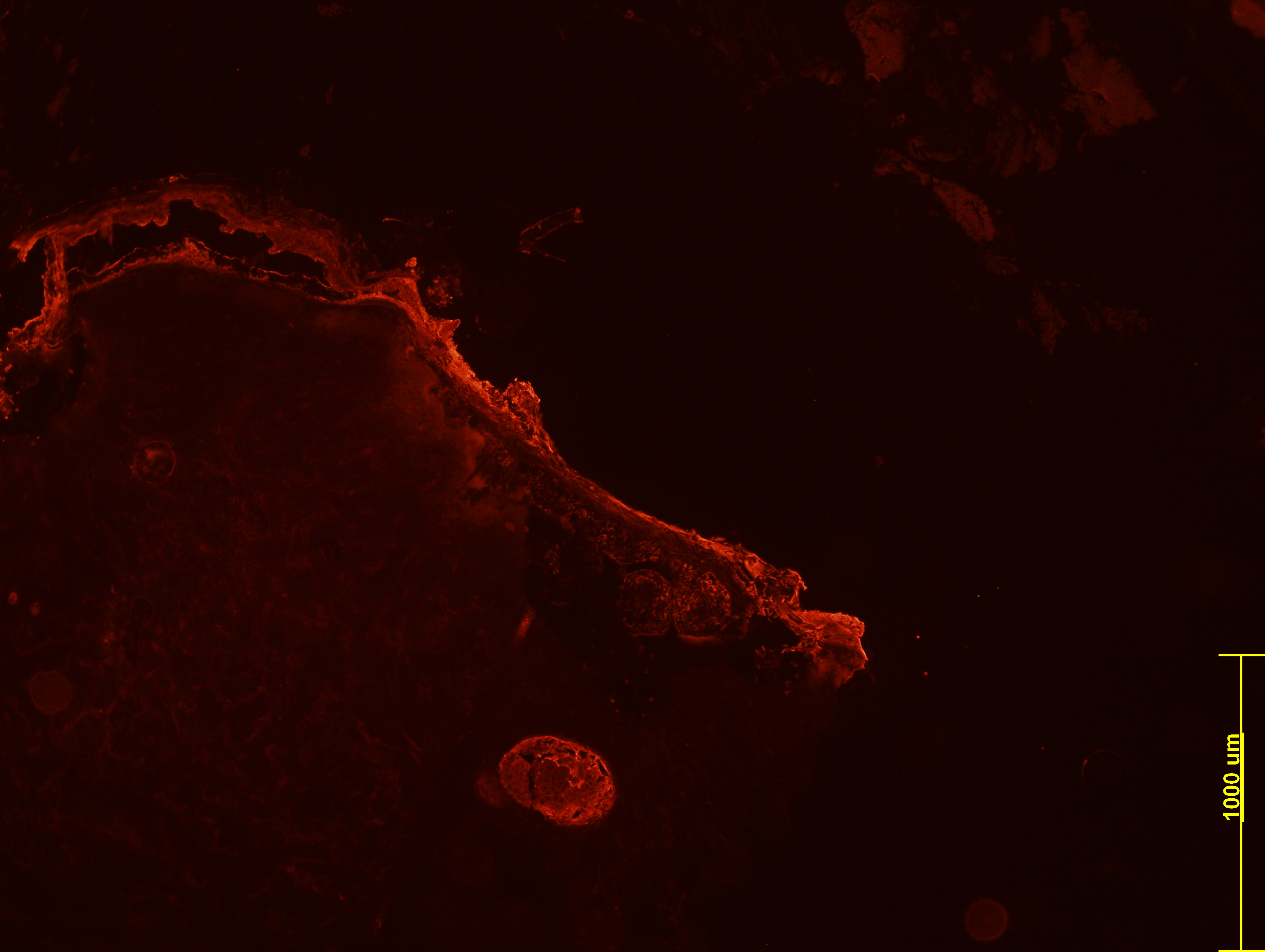

Supplement: S1 Imageset — (ZIP) [file pone.0128093.s004.zip › Immunos/MC/131009 mc d21 4.2 4x.jpg]

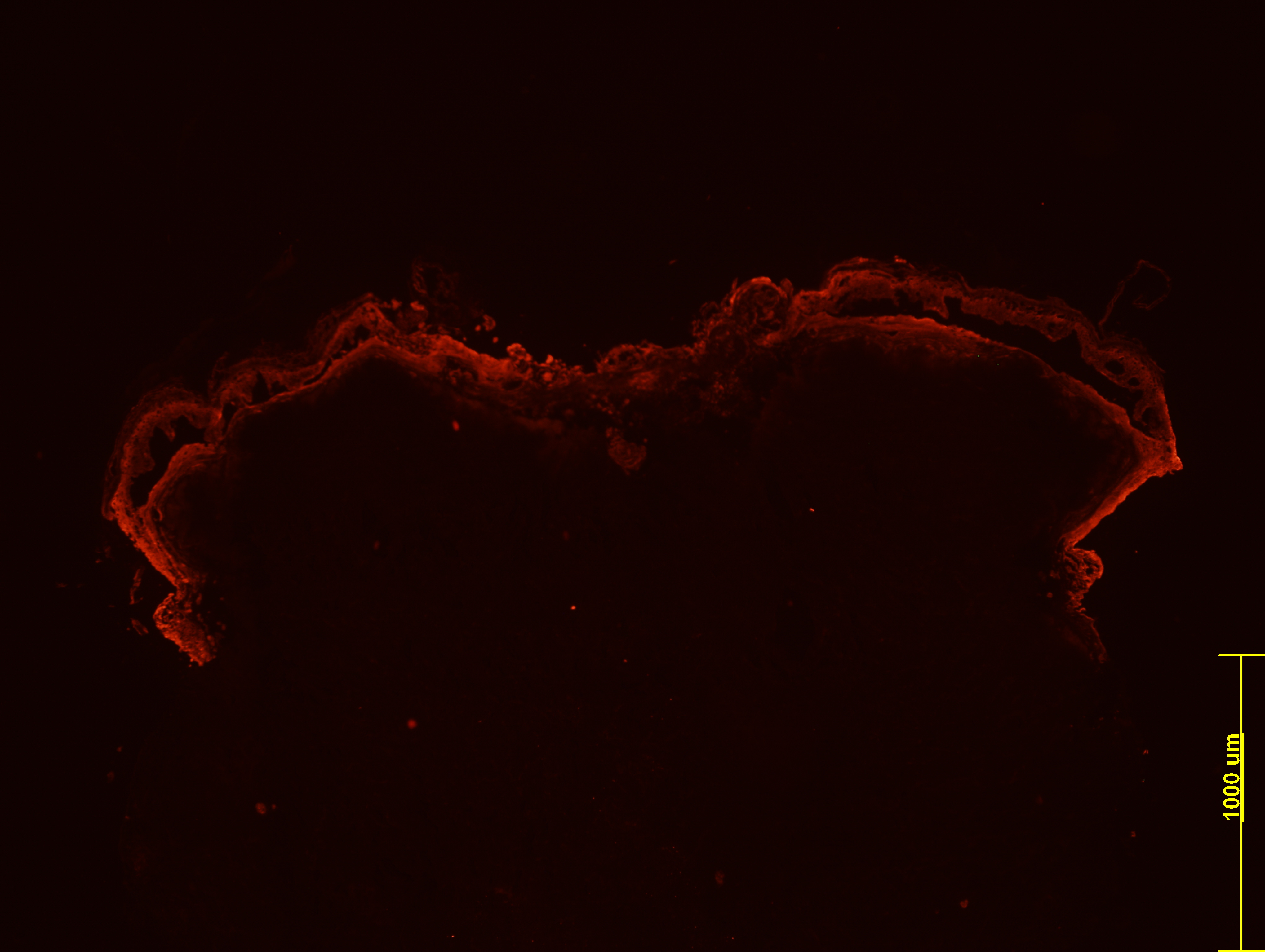

Supplement: S1 Imageset — (ZIP) [file pone.0128093.s004.zip › Immunos/MC/131009 mc d21 4.3 4x.jpg]

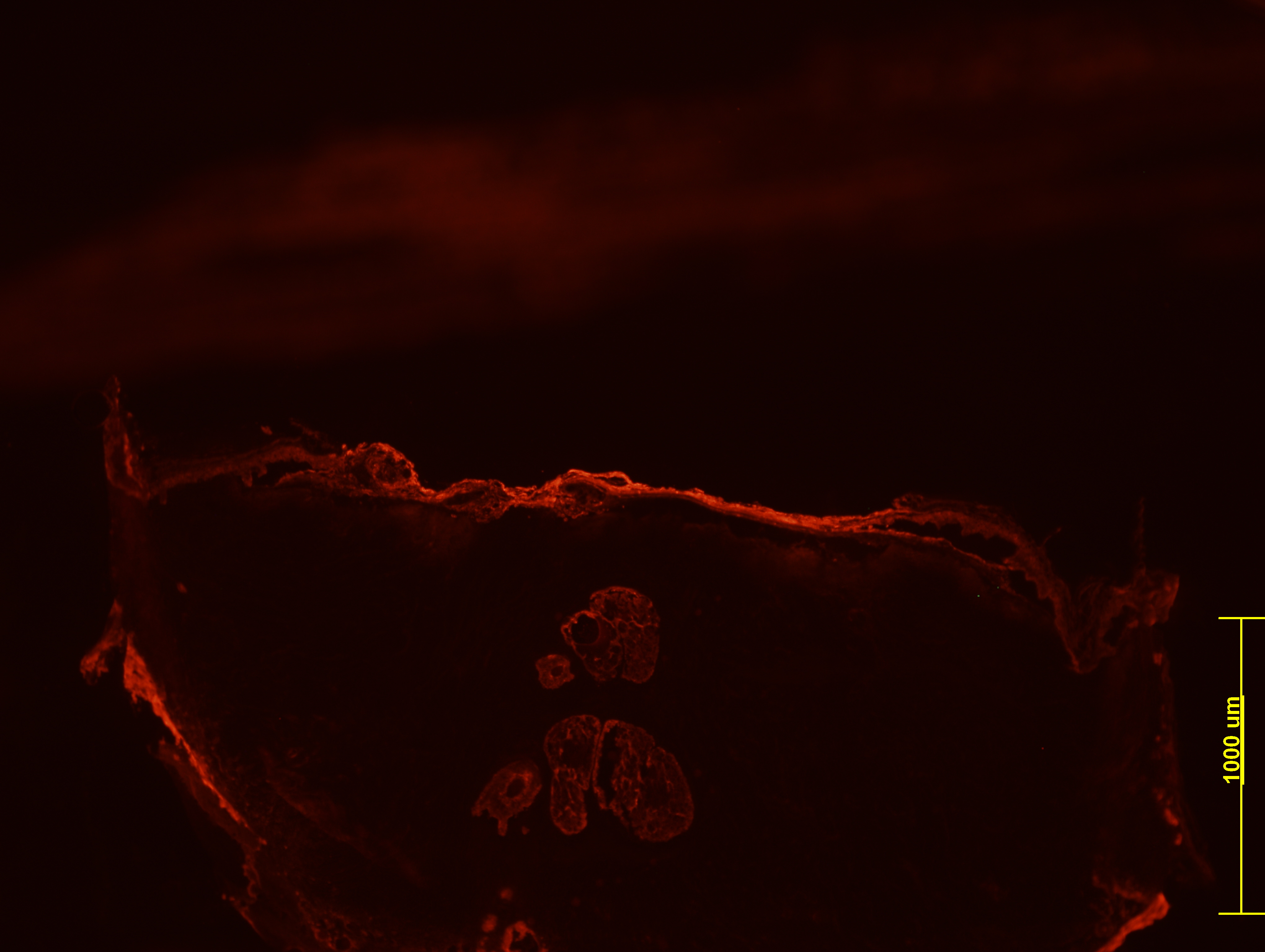

Supplement: S1 Imageset — (ZIP) [file pone.0128093.s004.zip › Immunos/MC/131009 mc d21 5.1 4x.jpg]

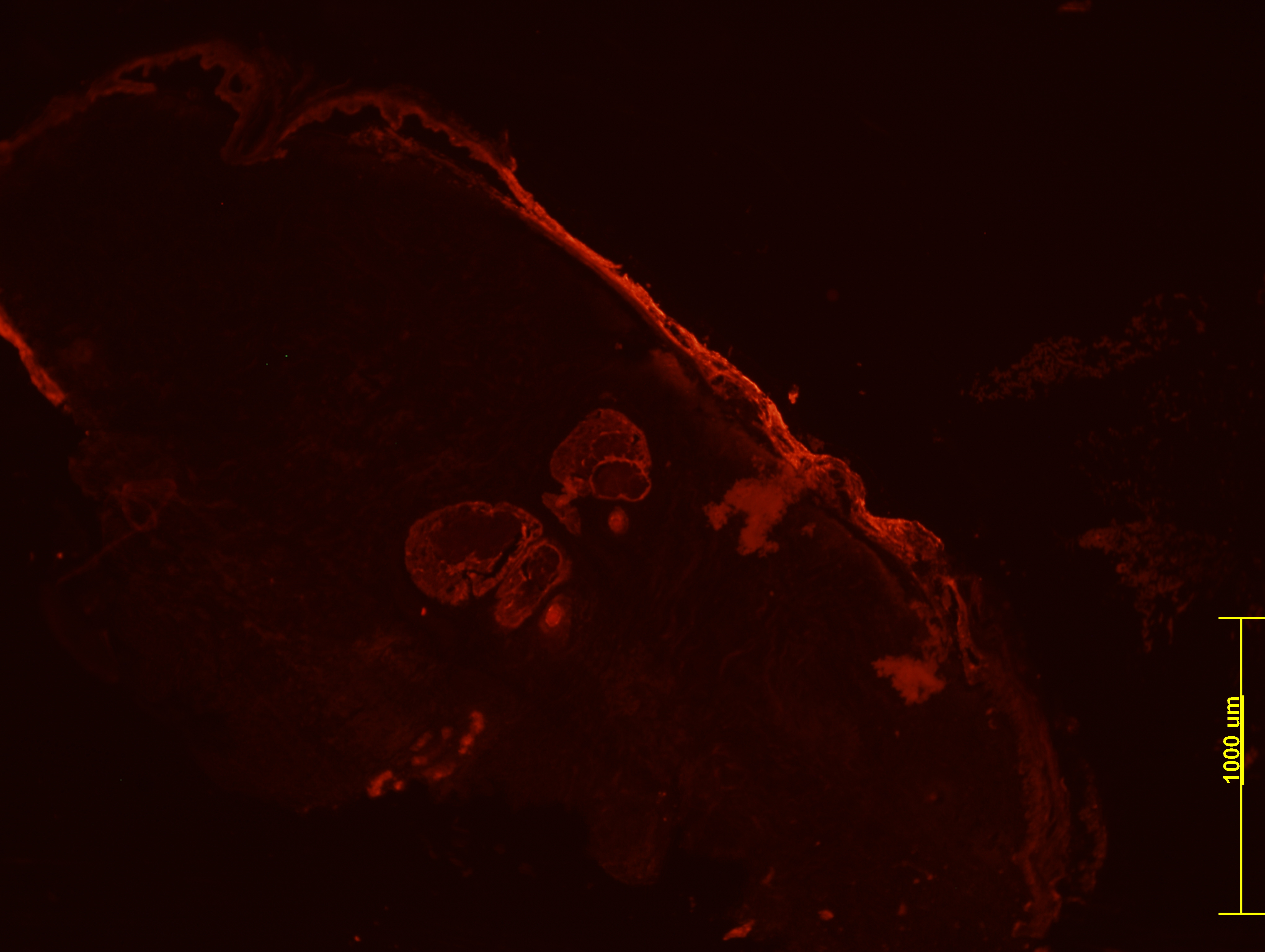

Supplement: S1 Imageset — (ZIP) [file pone.0128093.s004.zip › Immunos/MC/131009 mc d21 5.2 4x.jpg]

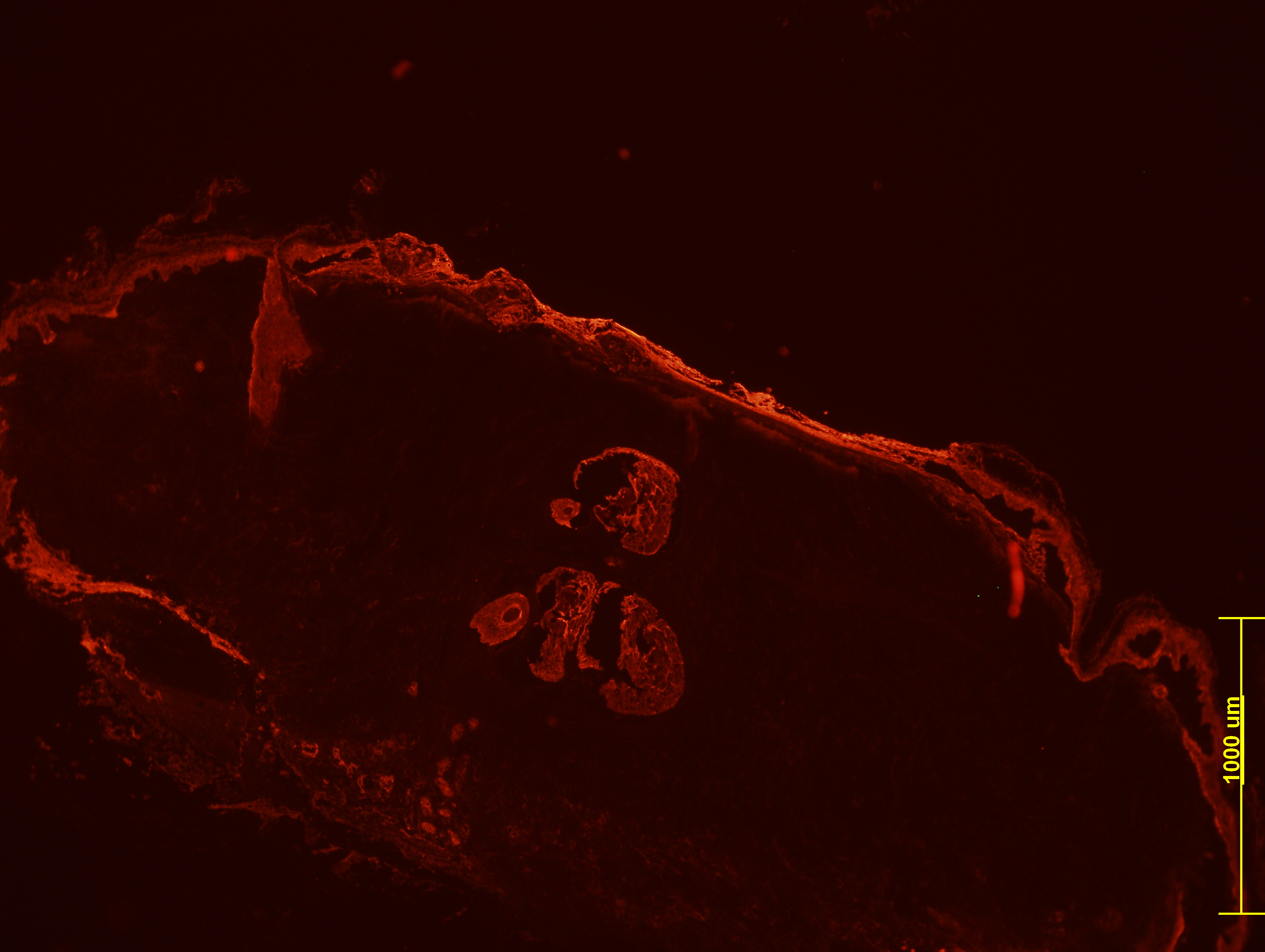

Supplement: S1 Imageset — (ZIP) [file pone.0128093.s004.zip › Immunos/MC/131009 mc d21 5.3 4x.jpg]

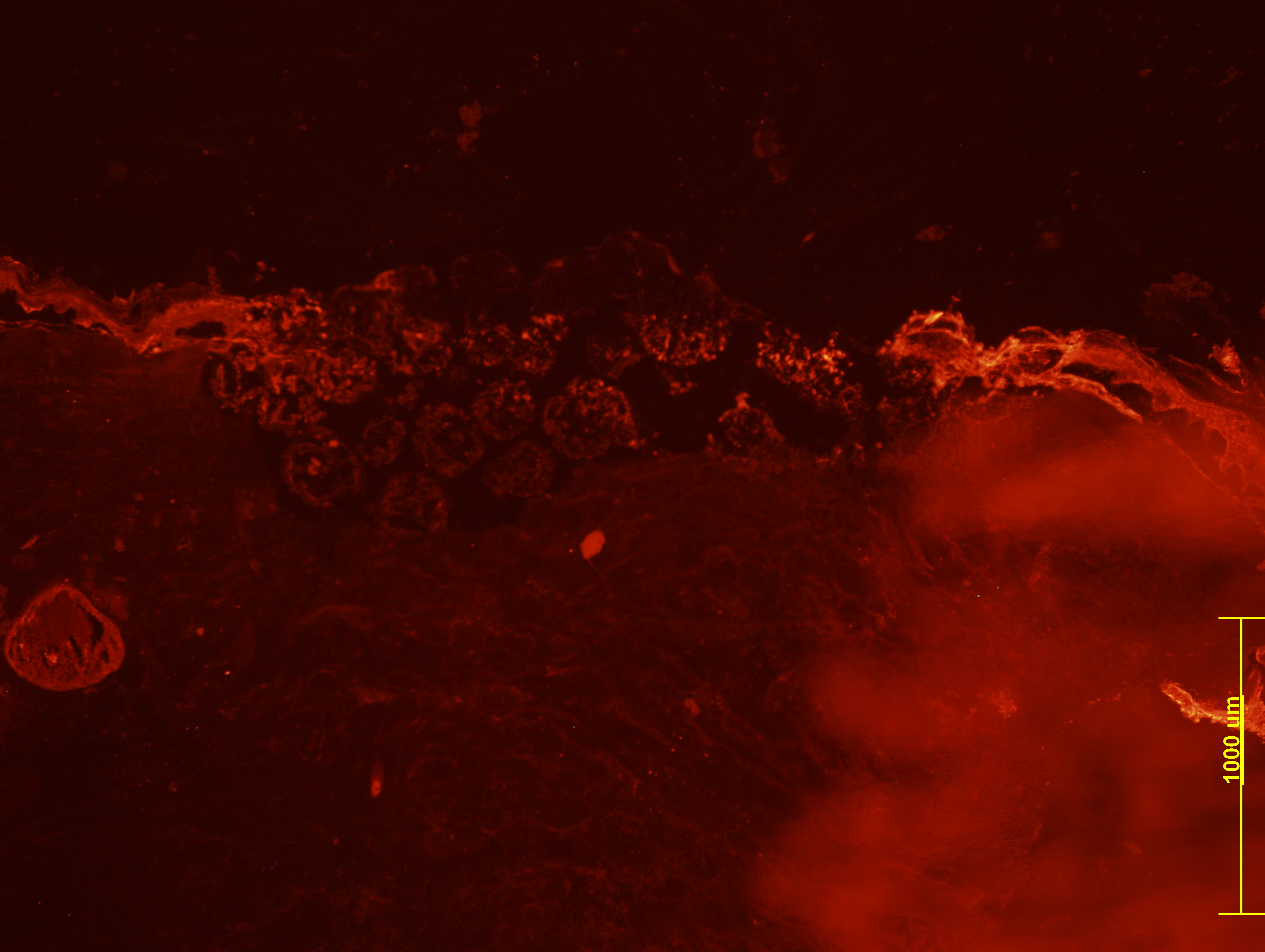

Supplement: S1 Imageset — (ZIP) [file pone.0128093.s004.zip › Immunos/MC/131009 mc d21 6.1 4x.jpg]

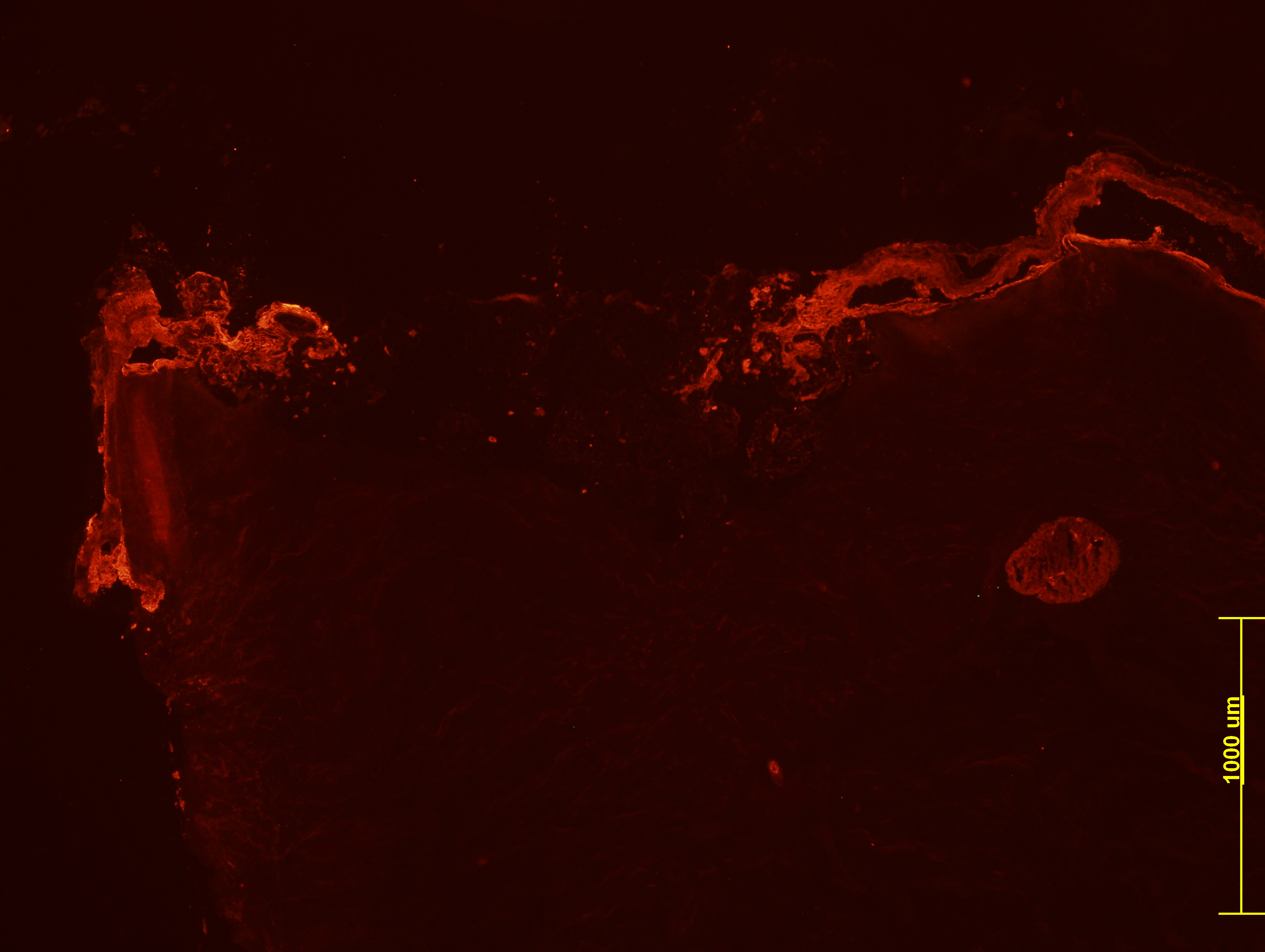

Supplement: S1 Imageset — (ZIP) [file pone.0128093.s004.zip › Immunos/MC/131009 mc d21 6.2 4x.jpg]

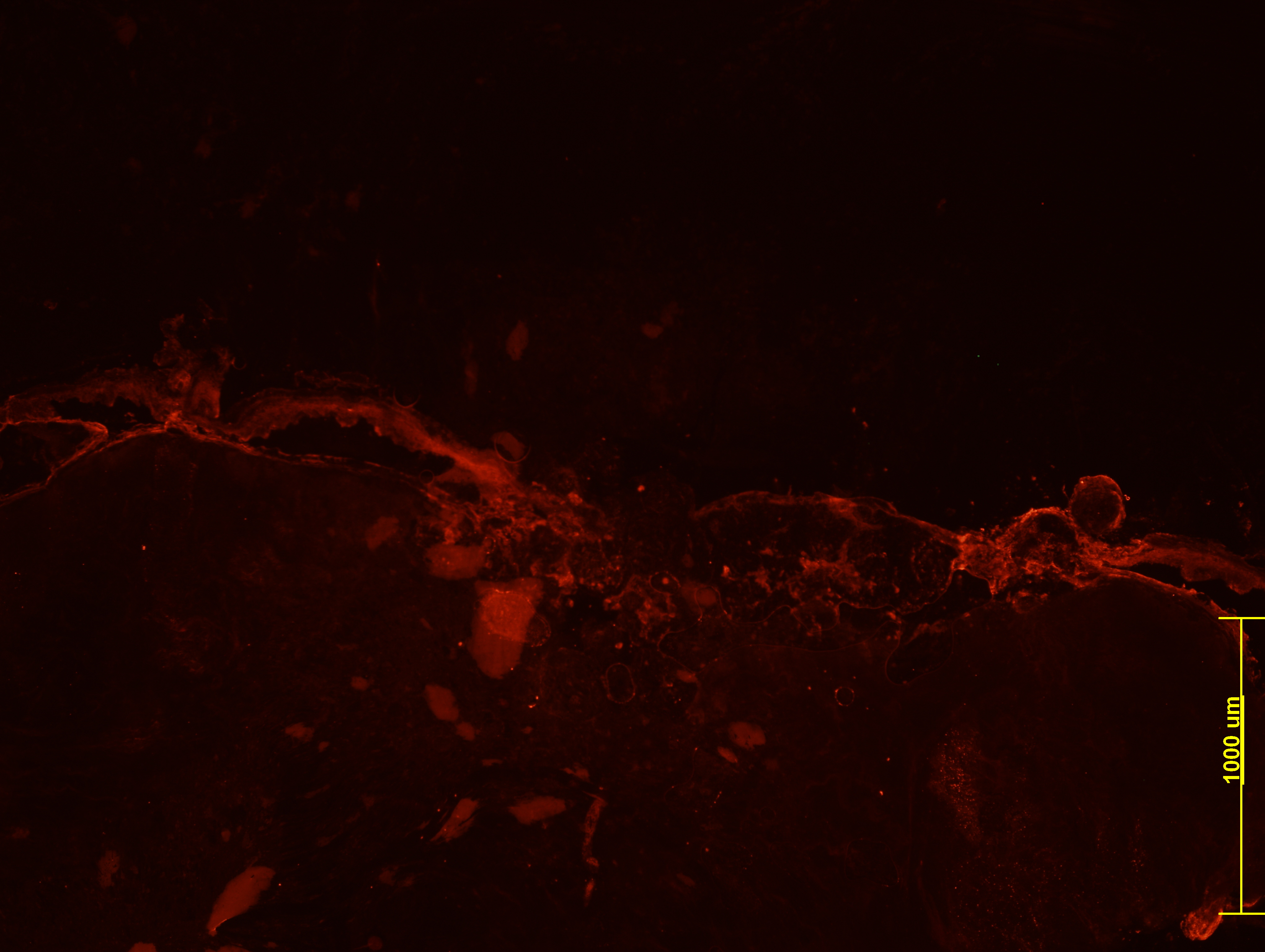

Supplement: S1 Imageset — (ZIP) [file pone.0128093.s004.zip › Immunos/MC/131009 mc d21 6.3 4x.jpg]
